# Boron Compounds Exhibit Protective Effects against Aluminum-Induced Neurotoxicity and Genotoxicity: In Vitro and In Vivo Study

**DOI:** 10.3390/toxics10080428

**Published:** 2022-07-28

**Authors:** Hasan Turkez, Serkan Yıldırım, Elvan Sahin, Mehmet Enes Arslan, Bugrahan Emsen, Ozlem Ozdemir Tozlu, Gonca Alak, Arzu Ucar, Abdulgani Tatar, Ahmet Hacimuftuoglu, Mevlut Sait Keles, Fatime Geyikoglu, Muhammed Atamanalp, Fatih Saruhan, Adil Mardinoglu

**Affiliations:** 1 Department of Medical Biology, Faculty of Medicine, Atatürk University, 25240 Erzurum, Turkey; hturkez@atauni.edu.tr; 2 Department of Pathology, Faculty of Veterinary, Atatürk University, 25240 Erzurum, Turkey; syildirim@atauni.edu.tr; 3 Department of Histology and Embryology, Faculty of Medicine, Sakarya University, 54050 Sakarya, Turkey; eozbek@sakarya.edu.tr; 4 Department of Molecular Biology and Genetics, Faculty of Science, Erzurum Technical University, 25050 Erzurum, Turkey; enes.aslan@erzurum.edu.tr (M.E.A.); ozlem.ozdemir@erzurum.edu.tr (O.O.T.); 5 Department of Biology, Kamil Özdağ Faculty of Science, Karamanoğlu Mehmetbey University, 70200 Karaman, Turkey; bemsen@kmu.edu.tr; 6 Department of Aquaculture, Faculty of Fisheries, Atatürk University, 25240 Erzurum, Turkey; galak@atauni.edu.tr (G.A.); arzuucar@atauni.edu.tr (A.U.); mataman@atauni.edu.tr (M.A.); 7 Department of Medical Genetics, Medical Faculty, Atatürk University, 25240 Erzurum, Turkey; abdulgani@atauni.edu.tr; 8 Department of Medical Pharmacology, Medical Faculty, Atatürk University, 25240 Erzurum, Turkey; ahmeth@atauni.edu.tr (A.H.); saruhanf@atauni.edu.tr (F.S.); 9 Department of Biochemistry, Medical Faculty, Uskudar University, 34664 Istanbul, Turkey; mevlutsait.keles@uskudar.edu.tr; 10 Department of Biology, Faculty of Arts and Sciences, Atatürk University, 25240 Erzurum, Turkey; fgeyik@atauni.edu.tr; 11 Science for Life Laboratory, KTH-Royal Institute of Technology, 114 28 Stockholm, Sweden; 12 Centre for Host-Microbiome Interactions, Faculty of Dentistry, Oral & Craniofacial Sciences, King’s College London, London WC2R 2LS, UK

**Keywords:** boron compounds, aluminum chloride, genotoxicity, cytotoxicity, oxidative status, neurotoxicity, hematotoxicity, histopathology, brain, blood

## Abstract

Genetic, neuropathological and biochemical investigations have revealed meaningful relationships between aluminum (Al) exposure and neurotoxic and hematotoxic damage. Hence, intensive efforts are being made to minimize the harmful effects of Al. Moreover, boron compounds are used in a broad mix of industries, from cosmetics and pharmaceuticals to agriculture. They affect critical biological functions in cellular events and enzymatic reactions, as well as endocrinal and mineral metabolisms. There are limited dose-related data about boric acid (BA) and other boron compounds, including colemanite (Col), ulexite (UX) and borax (BX), which have commercial prominence. In this study, we evaluate boron compounds’ genetic, cytological, biochemical and pathological effects against aluminum chloride (AlCl_3_)-induced hematotoxicity and neurotoxicity on different cell and animal model systems. First, we perform genotoxicity studies on in vivo rat bone marrow cells and peripheric human blood cultures. To analyze DNA and chromosome damage, we use single cell gel electrophoresis (SCGE or comet assay) and micronucleus (MN) and chromosome aberration (CA) assays. The nuclear division index (NDI) is used to monitor cytostasis. Second, we examine the biochemical parameters (superoxide dismutase (SOD), catalase (CAT), glutathione peroxidase (GSH-Px), malondialdehyde (MDA), total antioxidant capacity (TAC) and total oxidative status (TOS)) to determine oxidative changes in blood and brain. Next, we assess the histopathological alterations by using light and electron microscopes. Our results show that Al increases oxidative stress and genetic damage in blood and brain in vivo and in vitro studies. Al also led to severe histopathological and ultrastructural alterations in the brain. However, the boron compounds alone did not cause adverse changes based on the above-studied parameters. Moreover, these compounds exhibit different levels of beneficial effects by removing the harmful impact of Al. The antioxidant, antigenotoxic and cytoprotective effects of boron compounds against Al-induced damage indicate that boron may have a high potential for use in medical purposes in humans. In conclusion, our analysis suggests that boron compounds (especially BA, BX and UX) can be administered to subjects to prevent neurodegenerative and hematological disorders at determined doses.

## 1. Introduction

Aluminum (Al) is the third most abundant metal in nature [1]. Due to the extensive use of Al and Al-containing materials in our daily life, particularly in the last decade, the impacts of Al-induced cellular and systemic toxicities on human and animal health are becoming increasingly alarming. Studies have shown that daily Al intake can vary depending on food types; the most Al-containing foods are cereals, wheat, fruits, vegetables, coffee and tea [2]. Furthermore, drugs to treat ulcers have Al in notable amounts. Antacids are among the types of medications that include various salts of Al, magnesium, and calcium as active ingredients. Antacid drug abuse can cause the accumulation of Al and systemic metal toxicity [3,4]. It is known that Al accumulates in several organs, including the liver, kidney and brain [5]. The accumulation of Al in the brain leads to the development of neurological disorders. Al-containing compounds induced glial activation and increased inflammatory cytokines and amyloid precursor proteins within different brain regions [6]. Acute or chronic exposure to Al causes structural, physiological, and neurochemical alterations in the nervous system. Recent findings revealed that Al-induced neurotoxicity is enhanced by the accumulation of β-amyloid peptide, considered a fundamental cause of Alzheimer’s disease (AD) and related neurological disorders [7,8]. Besides, Al is regarded as a main environmental risk factor for variegated alterations in the peripheral blood and hemogenic system. It has been reported that the Al-induced hematological changes alter heme biosynthesis, decrease the lifetime period and osmotic resistance of red blood cells and affect the defensive ability of white blood cells [9].

**Figure 1:**
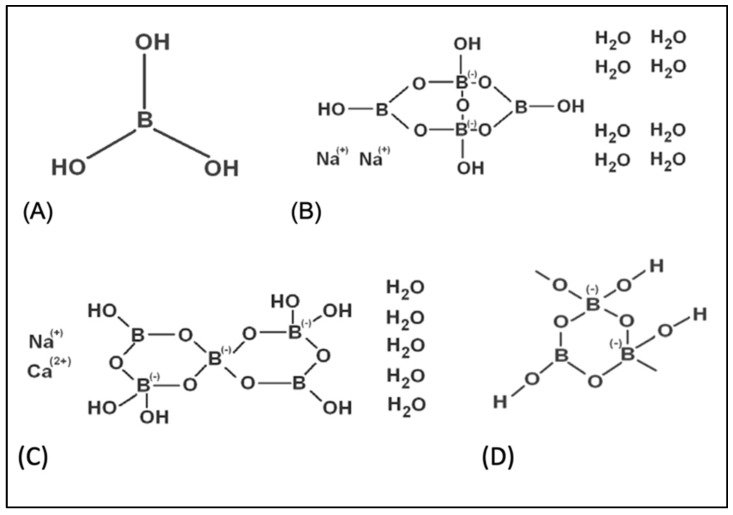
The chemical structures of tested boron compounds. (**A**) boric acid, (**B**) borax, (**C**) ulexite, (**D**) colemanite.

Boron (B) is used in over 400 areas, including the fertilizer, glass, pharmaceutical, chemical, nuclear, automobile and spacecraft industries. Hence, B is considered a strategic element worldwide, whose application and usage areas are increasing daily with technological developments and recent scientific data about the physicochemical and biological properties of boron-containing compounds. B commonly occurs in nature as borates such as boric acid (BA), borax (BX), ulexite (Ule) and colemanite (Col) (Figure 1). BA is the most common form of the borates and it has been shown that the pharmacokinetics of BA in humans and rodents are quite similar [10]. BA and BX, two widespread forms of borates, are used in tablets, effervescent powders, capsules, chewable tablets, and several liquid formulations as food supplements [11]. B is an essential microelement for plants and is necessary for their optimum development. However, the necessity of B for humans and animals has not been definitively reported, despite the multiplexed scientific evidence. B is mentioned as a probably essential element by the World Health Organization (WHO) [12]. B and B-containing compounds positively impact bone growth and brain functions, modulate arthritic symptoms and improve the effect of hormones [13]. In addition to these positive health impacts, boron-based formulations exhibit anticancer, antiviral, anti-inflammatory, anti-mutagenic, antibacterial and antifungal properties [14,15]. Moreover, it has been reported that different borates, including BA, BX, Ule and Col support the antioxidant capacity of human blood cells [16]. Recent investigations indicated that the supplementation of B led to the enhancement of antioxidant defense mechanisms in cells or organisms and exerted a good potency of protection against oxidative damage [17,18,19].

The toxic effects of Al are mainly through systemic peroxidative damage and the intracellular accumulation of reactive oxygen species (ROS) [20]. Inhibition of antioxidant enzyme activities in the brain and blood suggested the pro-oxidant status of this metal. The promotion of oxidative stress in tissues has been linked to the direct effect of Al on enzymes or their synthesis [21,22,23]. Although the mechanisms of neurotoxic and hematotoxic damage by AI have not been fully explained, several antioxidants or featured antioxidant compounds, including vitamin E, melatonin and selenium, were found to counteract Al-induced oxidative damage [24,25,26]. Hence, examining antidotes against the toxic effects of Al is especially and crucially important [27]. The supplementation of natural substances with free radical scavenging activity may protect the cells or organisms from the harmful effects of Al [28].

Based on toxicological considerations related to the mechanism of Al toxicity and the beneficial biological properties of B, we tested a hypothesis that B-containing compounds might be protective against neurotoxic agents in vitro and in vivo studies. Given the cytoprotective potential of boron compounds and the limited number of investigations regarding its mechanisms of action, we evaluated the in vitro and in vivo potential neuroprotective and hemato-protective role of four different commercially important borates—BA, BX, Ule and Col—against Al-induced damage. Therefore, human blood and primary rat cortical neurons (PRCNs) were used to monitor in vitro hematotoxicity and neurotoxicity assessments. In vitro hemato-protective and protective potential by the boron compounds against Al-induced toxicity were determined. The MTT assay was performed to determine the cell viability of both cells in culture. In addition, we decided on biochemical parameters involving superoxide dismutase (SOD), catalase (CAT), glutathione peroxidase (GSH-Px), malondialdehyde (MDA), total antioxidant capacity (TAC) and total oxidative stress (TOS) levels. The effects of Al and boron compounds on in vitro genetic damage were assessed using three different genotoxicity endpoints, including single cell gel electrophoresis (SCGE, comet assay) and micronucleus (MN) and chromosome aberration (CA) assays. Sprague-Dawley rats were used in vivo studies. Al and boron compounds were applied chronically to rats separately and in combination. Brain histopathology was evaluated using light and electron microscopy in addition to in vivo biochemical and genetic evaluations on the animal brain and blood samples.

## 2. Materials and Methods

### 2.1. Cell Cultures

The peripheral human blood cells were cultured according to the protocol as previously described [29]. Blood samples (30 mL) were collected from healthy individuals (*n* = 6) between the ages of 24 and 30 (26.0 ± 2.3) who were nonsmoking, non-alcoholic and not under drug therapy, as well as not exposed to any toxic agents due to their professions. The heparinized blood samples (0.6 mL, final concentration of heparin was 40 IU/mL) were cultured in 7 mL of culture medium (Chromosome Medium B, Sigma-Aldrich, St. Louis, MO, USA) with phytohemagglutinin (5 mg/mL, Biochrom, Cambridge, UK) [30].

Primary rat cortical neurons (PRCNs) were set up using rat fetuses as previously described [31]. For this, eight newborn rats were sacrificed via a sterile decapitation process. After removal of skin and skull, meninges were separated; then, the frontal cortex was isolated. Cortex particles were kept in Hanks’ Balanced Salt Solution (HBSS, 5 mL) and macromerotomy was done. The occurred tissue composition was pulled into a syringe, placed into a media containing HBSS plus Trypsin-EDTA (0.25% trypsin–0.02% EDTA) and chemical decomposition was achieved. After the addition 120 U/mL of DNase type 1, the existing solution was centrifuged for 3 min and at 800 rpm. The obtained single cells after physical and chemical decomposition steps were divided into flasks coated with poly-Dlysine. These flasks were kept in the incubator (5% CO_2_, 37 °C) at the ventile position. The medium of the flasks was changed with a fresh medium every 3 days until the cells were branched and had reached a certain maturity.

In vitro neurotoxicity experiments were performed eight days later using 150 µL of the mixture, which was added into each well of 96-well plates and incubated (37 °C, 5% CO_2_) in the incubator for one week. The mix of neurobasal medium + B27 was added into the wells containing cells at a rate of ½ of cell volume. We waited until neuronal cells in the base of the plate showed branching so they could be seen under a microscope. These experiments were performed at the Medical Experimental Animal Research Centre in Atatürk University (ATADEM, Erzurum, Turkey). The ethical committee of Atatürk University approved the experimental protocol of this study (B.30.2.ATA.0.70/115).

### 2.2. In Vitro Applications

BA, BX, Col and UX compounds were purchased from Eti Mine Works (Ankara, Turkey) and applied to the cell cultures in a wide range of concentrations from 0.01 mg/L to 20 mg/L. Stock solutions of the compound were prepared in culture medium as 1 g/L and diluted in culture medium to reach the desired concentrations. Aluminum chloride (AlCl_3_) was purchased from Sigma-Aldrich (St. Louis, MO, USA) and applied to the cell cultures at a final concentration of 20 mg/L. Co-applications were performed for 20 mg/L of AlCl_3_ and boron compounds (0.01 mg/L to 20 mg/L.) simultaneously. Each primary human whole blood and PRCNs without boron compounds and Al was studied as a control group. Triton X-100 (1%) was used as a positive control in determining cell viability rates.

### 2.3. Cytotoxicity Testing

Cell viability rates were determined using commercially available kits (MTT Cell Proliferation Kit, Cayman, Ann Arbor, MI, USA) after applications with boron compounds and Al for 72 h. The viability of cells was evaluated by spectrophotometric measurement formazan amounts from MTT. At the end of the culture period of 72 h, the cell cultures were incubated with 0.6 mg/mL MTT for 2 h at 37 °C. Then, the available medium was eliminated using the spinning technique and the formazan was dissolved in DMSO. The optical density was measured at 570 nm using an ELISA reader.

### 2.4. Biochemical Assays

#### 2.4.1. Superoxide Dismutase (SOD) Activity

The SOD activity in the medium released from the peripheral human blood and PRCNs cells is similar as described previously by [31,32]. In brief, 9.13 g xanthine (0.3 mM) was dissolved in 200 mL distilled water (Solution 1). 25 mg ethylenediaminetetraacetic acid (EDTA) was dissolved in 100 mL distilled water (Solution 2); 12.3 g nitroblue tetrazolium (NBT) was dissolved in 100 mL distilled water (Solution 3); 2.54 g Na_2_CO_3_ (0.4 M) was dissolved in 60 mL distilled water (Solution 4); 30 mg bovine serum albumin was dissolved in 30 mL distilled water (Solution 5). Solutions 1–5 (test reagent) were stored at +4 °C. 30 μL xanthine oxidase was diluted with 1 mL of 2 M (NH_4_)_2_SO_4_. For the sample tube, the test reagent (2.5 mL), sample (0.5 mL) and xanthine oxidase (50 μL) were mixed and incubated in a water bath at 25 °C for 25 min. The percentage (%) inhibition and enzyme activities caused by the SOD enzyme in the sample were calculated as follows:

$$\mathrm{Inhibition}\;\left(\%\right)=100\times \frac{\mathrm{Blank}\;\mathrm{absorbance}-\mathrm{Sample}\;\mathrm{absorbance}}{\mathrm{Blank}\;\mathrm{absorbance}}$$

$$\mathrm{SOD}\;\left(\frac{U}{\mathrm{mL}}\right)=\frac{\mathrm{Inhibition}\;\left(\%\right)}{50\times 0.5\;\mathrm{mL}}$$

#### 2.4.2. Catalase (CAT) Activity

CAT activity in treated and untreated cells was determined by a previously described protocol [33,34]. In brief, 2.8 g of EDTA was added to the phosphate buffer (pH 7.0). 30% H_2_O_2_ was added on 200 mL of phosphate buffer until the absorbance was 0.5 at 240 nm wavelength. Phosphate buffer plus H_2_O_2_ (2.99 mL) and medium sample (0.01 mL) were mixed in a quartz cuvette and read at room temperature at 240 nm wavelength 2 times at 15 s intervals. CAT activity was calculated as follows:

$$\mathrm{CAT}\;\left(\frac{U}{g}\mathrm{Hb}\right)=0.153\times \frac{\log A1}{\log A2}$$

#### 2.4.3. Glutathione Peroxidase (GSH-Px) Activity

GSH-Px activity in medium samples obtained from cultures was measured with the method previously suggested by [35,36]. In this method, 50 mg GSH (150 mM) was dissolved in 1 mL of phosphate buffer; 50.4 mg NADPH (10 mM) was dissolved in 6 mL of the buffer; GSH reductase (10 μL) was added to the experimental medium; 25 μL 30% H_2_O_2_ (2 mM) was dissolved in 5 mL buffer. For the sample tube, phosphate buffer (205 mL), GSH (100 µL), NADPH (100 µL), sample (20 µL) and H_2_O_2_ (100 µL) were mixed and incubated in a water bath at 37 °C for 30 min. The GSH-Px activity was calculated as follows:

$$\mathrm{GSH}-\mathrm{Px}\;(U/L)=\mathrm{Dilution}\;\mathrm{coefficient}\;\times \;\frac{\Delta A/t\;\times \;10^{6}}{\varepsilon }$$

ΔA/t: absorbance change per minute; ε: molar absorptivity coefficient of NADPH at 340 nm (6.22 × 103).

#### 2.4.4. Malondialdehyde (MDA) Analysis

MDA levels in cultured cells were determined by the method previously reported by [37,38]. In brief, saline buffered with phosphate buffer was prepared. 30 g trichloroacetitic acid (TCA) was dissolved in water and its volume was completed to 100 mL; 37.224 g EDTA was dissolved in water and its volume was made up to 1 L. 1 g of thiobarbituric acid (TBA) was dissolved in 0.5 N NaOH; 0.088% butylated hydroxytoluene (BHT) was dissolved in 10 mL of ethanol. For the sample tube, phosphate buffer (800 μL), sample (200 μL), BHT (25 μL), and TCA (50 μL) were kept at 20 °C by vortexing. The solution was allowed to melt during use and then the supernatant was obtained by centrifugation at 2000 rpm for 15 min. The supernatant (1 mL), EDTA (75 μL), and TBA (250 μL) were mixed and incubated in a water bath at 95 °C for 15 min. At the end of the incubation period, the samples were kept at room temperature. Spectrophotometric readings were carried out at 532 and 600 nm wavelengths. MDA activity was calculated as follows:

$$\mathrm{MDA}\;\left(µ\mathrm{mol}/L\right)=\mathrm{Dilution}\;\mathrm{coefficient}\times \frac{\mathrm{Absorbance}\times 10^{6}}{1.52\times 10^{5}}$$

#### 2.4.5. Total Antioxidant Capacity (TAC) and Total Oxidative Stress (TOS) Levels

The TAC method was based on the determination of the antioxidant capacity to inhibit the formation of the 2,2′-azino-bis (3-ethylbenzothiazoline-6-sulfonic acid) (ABTS) radical. TOS detection method was based on spectrophotometric measurement of colored complexes formed by iron ions with chromogen in acidic mediums. In the detection process, commercially available kits (Rel Assay Diagnostics, Gaziantep, Turkey) were used [39].

### 2.5. Genotoxicity Testing on Human Blood Cultures

#### 2.5.1. In Vitro CA Assay

Al (20 mg/L) and each boron compound (5 and 10 mg/L) were applied to human lymphocyte cultures for 72 h. The colchicine solution (0.02 μg/mL of) was added into the culture 2 h prior to harvesting. After performing hypotonic treatment (0.075 M KCl) and fixation (methanol + acetic acid) three times, cells were centrifugated (900 rpm, 10 min) for harvesting. The slides were prepared from each fixed-cell suspension and kept for air-drying for 24 h. Then, the preparations were stained due to the Giemsa protocol for 15 min. For each treatment, at least 25 well-spread metaphases per culture were scored for chromosome aberration analysis in six different repetitions. The aberrations (chromatid or chromosome gap and chromatid or chromosome break) were classified according to the previously suggested criteria of Environmental Health Criteria 46 for environmental monitoring in human populations [40].

#### 2.5.2. In Vitro MN Assay

After 44 h from the beginning of the incubation, cytochalasine-B was added into cultures with a final concentration of 6 μg/mL. At the end of 72 h, the tubes were centrifuged (900 rpm, 10 min). Centrifugation procedures, the addition of hypotonic and fixation solutions, and smears were performed using the protocol previously given for CA assay. The preparations were kept in Giemsa dye and after dyeing, they were passed through distilled water again and left to dry at room temperature. The preparations were examined under a light microscope with an immersion (×100) objective. At least 1000 binucleated cells were examined in each culture for the presence of one, two or more micronuclei [41].

#### 2.5.3. In Vitro SCGE Assay

SCGE or comet assay was performed due to a minor modification of the previously reported protocol [42]. In sum, after the application of coverslips, the prepared slides were allowed to gel at 4 °C for 60 min. Then, the slides were immersed in NaCl, EDTA, Tris, dimethyl sulphoxide and Triton X-100 containing cold lysing solution. After lysing process, the slides were taken to alkali treatment with NaOH (300 mM) and EDTA (1 mM), followed by electrophoresis (300 mA and 25 V) and neutralization (0.4 M Tris) phases. After these steps, the slides were stained with ethidium bromide (20 µg/mL). DNA damage was observed by using a fluorescence microscope (×100, Nikon Eclips E6600, Tokyo, Japan). Finally, a total of 100 cells were scored for each slide to calculate by multiplying the number of cells considered for each grade of damage between grades 1 and 4. Hence, the maximum damage score of 400 was applied [43].

### 2.6. Experimental Animals

Male Sprague-Dawley rats weighing 280–300 g were utilized and housed at a regulated temperature of 22 ± 2 °C and a controlled humidity of 5% on a 12 h light/dark cycle. The animal experiments were performed at the Medical Experimental Animal Research Centre in Atatürk University (ATADEM, Erzurum, Turkey). The ethical committee of Atatürk University approved the experimental protocol of this study (B.30.2.ATA.0.70/115), which was carried out in line with the National Institutes of Health Guide for the Care and Use of Laboratory Animals.

### 2.7. Treatments with Al, BA, BX, Col and UX

After a week of acclimating to laboratory settings, rats were randomly divided into 18 groups (*n* = 6 in each group) as follows: (1) control group (physiological saline), (2) Al-treated (injected intraperitoneally) group for 30 days (as AlCl_3_, 4.2 mg/kg b.w. intraperitoneal (IP) injection), (3) BA-treated group for 30 days (3.25 mg/kg b.w. IP), (4) BA-treated group for 30 days (6.5 mg/kg b.w. IP), (5) BX-treated group for 30 days (3.25 mg/kg b.w. IP), (6) BX-treated group for 30 days (6.5 mg/kg b.w. IP), (7) Col-treated group for 30 days (3.25 mg/kg b.w. IP), (8) Col-treated group for 30 days (6.5 mg/kg b.w.), (9) UX-treated group for 30 days (3.25 mg/kg b.w. IP), (10) UX-treated group for 30 days (6.5 mg/kg b.w. IP), (11) co-application (IP) of 3.25 mg/kg BA and 4.2 mg/kg Al, (12) co-application of 6.5 mg/kg BA and 4.2 mg/kg Al, (13) co-application of 3.25 mg/kg BX and 4.2 mg/kg Al, (14) co-application of 6.5 mg/kg BX and 4.2 mg/kg Al, (15) co-application of 3.25 mg/kg Col and 4.2 mg/kg Al, (16) co-application of 6.5 mg/kg Col and 4.2 mg/kg Al, (17) co-application of 3.25 mg/kg UX and 4.2 mg/kg Al, (18) co-application of 6.5 mg/kg UX and 4.2 mg/kg Al. The application doses were selected due to our in vitro findings and previous literature data [2,44,45]. Rats were given rat chow and tap water ad libitum.

### 2.8. In Vivo Biochemical Assays

At the end of the experiment, brains tissue and serum samples were taken from animals under ether anesthesia in order to determine the oxidative alterations using different parameters, including activities of SOD, CAT and GSH-Px, as well as MDA, TAC and TOS levels, as described in part of the in vitro biochemical assays.

### 2.9. In Vivo Genotoxicity Testing

#### 2.9.1. In Vivo CA Assay

While applying the CA assay, the following steps were followed. Firstly, femurs of animals treated with Al and boron compounds were removed under anesthesia and one of them was used for the application of the CA method. The marrow to be removed from the femur was taken into a tube containing 2 mL of PBS solution and then centrifuged (1200 rpm, 10 min); the supernatant was discarded. Cells were kept in a hypotonic environment at 37 °C for 30 min and fixed in a methanol: acetic acid (3:1) solution. Preparations were kept at +4 °C for 24 h; then, they were stained with Giemsa dye and kept for analysis. Structural and numerical chromosomal aberrations were scored in at least 30 metaphases per animal [41,46]

#### 2.9.2. In Vivo MN Assay

The other femurs taken under ether anesthesia from animals treated with Al and boron compounds were used for the application of MN assay. The marrow to be removed from the femur was taken into a tube containing 2 mL of fetal bovine serum (FBS); then, the serum was removed by centrifuging the tubes (2000 rpm, 3 min). The pellet was dropped on the slides and after the drying step, it was fixed in methanol for 10 min. Following the fixation process, the Giemsa staining procedure was applied. 1000 polychromatic erythrocytes (PCE) from each animal were scored for the presence of MN formations [47].

#### 2.9.3. In Vivo SCGE Assay

The frequency of strand breaks in leukocyte DNA was determined by comet assay. Leukocyte suspension was spread on a microscope slide covered with low melting point agar gel and covered with a second layer of agar. By dipping the slide into a lysis solution containing a high concentration of salt, all cell contents except nuclear material were lysed. Then, cellular residues were removed by washing in buffer solution (pH: 8). When nucleoid DNA containing supercoil structure and containing a small amount of non-histone protein was kept in an alkaline medium, the supercoil structure was loosened and opened in the regions where there were chain breaks. After electrophoresis in an alkaline medium (pH > 13), the pH was neutralized and the sample was stained with ethidium bromide and examined under a fluorescence microscope. DNA damage was calculated by examining 100 nucleoid DNAs for each culture. comet formations were divided into five different groups according to the observed tail lengths and scored from zero to four (undamaged DNA: 0 points; maximum damaged 4 points). Thus, the total damage score obtained for 100 comets was determined to be between 0–400. All these processes were carried out in the dark in order to prevent environmental DNA damage [30,48].

### 2.10. Histopathological Examination

The dissected rat brains were fixed using formalin solution (10%) and embedded in paraffin. Sections of 5 µm thickness were procured at the level of the cortex and hippocampus regions of the rat brain. Then, the preparations were stained with hematoxylin and eosin and examined under a light microscope (Nikon Eclipse 600; Nikon Co., Tokyo, Japan) for histological examination [48,49]. Observed histopathological findings were evaluated as absent (-), very mild (+), mild (++), moderate (+++), severe (++++) and very severe (++++++), according to the severity and extent [50].

For electron microscopy, cortical and hippocampal tissue samples were fixed in 3% glutaraldehyde in 0.1 M phosphate buffer for 30 min at room temperature and postfixed in osmium tetraoxide (1%) for 1 h. After the fixation, the samples were dehydrated through a graded acetone series, and embedded in Araldite CY212. Araldite-embedded samples were cut into 1 μm thickness semithin sections using a microtome (Nova Ultratome, Stockholm, Sweden) and then toluidine blue staining procedure was performed. The obtained ultrathin sections (at 70–80 nm) were taken onto copper grids and contrasted using 2% uranyl acetate and 0.4% lead citrate. The ultrathin sections were examined under a transmission electron microscope (Jeol 100 SX; Jeol, Tokyo, Japan) [51].

### 2.11. Statistical Analysis

One-way ANOVA and Fisher’s least significant differences (LSD) tests were applied using SPSS (IBM SPSS Statistics 12; IBM Company, Armonk, NY, USA) to determine the mean damage score, MN, CA values, SOD, CAT and GSH-Px, enzyme activities, and MDA, TAC, and TOS levels compared to the control groups. The obtained results were interpreted considering the 0.05 significance level.

## 3. Results

The hematotoxic and neurotoxic effects of four different boron compounds (in 11 different concentrations) and Al were determined colorimetrically and quantitatively by using an MTT assay. Table 1 and Table 2 show the results of statistical comparisons of treated and untreated cultures. After application with Al (20 ppm), we found a statistically significant (*p* < 0.05) decrease in blood and PRCNs cultures compared to untreated culture (Table 1). Our findings also revealed that all tested boron compounds did not induce any cytotoxic damage on both human blood and PRCNs cells. Moreover, BA, BX, Col and UX provided different levels of protection against the toxicity of Al in precise concentration dependent manners (Table 2).

**Figure 2:**
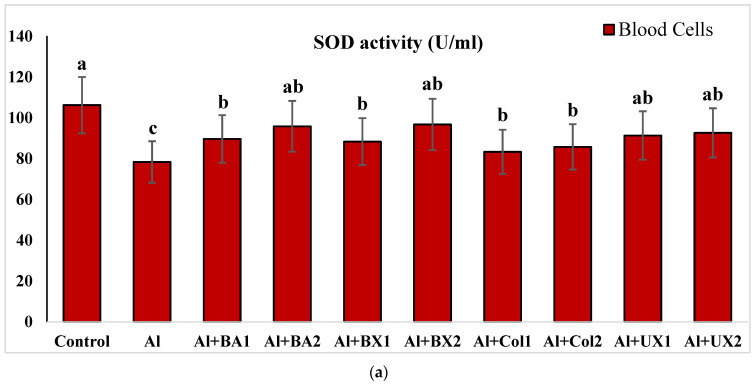

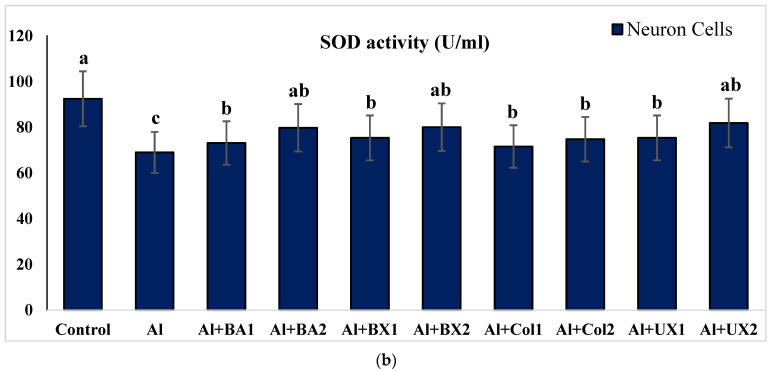
The effect of Al and boron treatments in (**a**) human blood and (**b**) PRCNs cells on SOD activity. (Control: untreated cultures; positive control as Al: 20 ppm AlCl_3_; BA1: 5 ppm boric acid; BA2: 10 ppm boric acid; BX1: 5 ppm borax; BX2: 10 ppm borax; Col1: 5 ppm colemanite; Col2: 10 ppm colemanite; UX1: 5 ppm ulexite; UX2: 10 ppm ulexite; values in the same column with different letters are significantly different (*p* < 0.05; Duncan’s test). (Each superscript letter (a, b, c) represents statistically similar groups).

**Figure 3:**
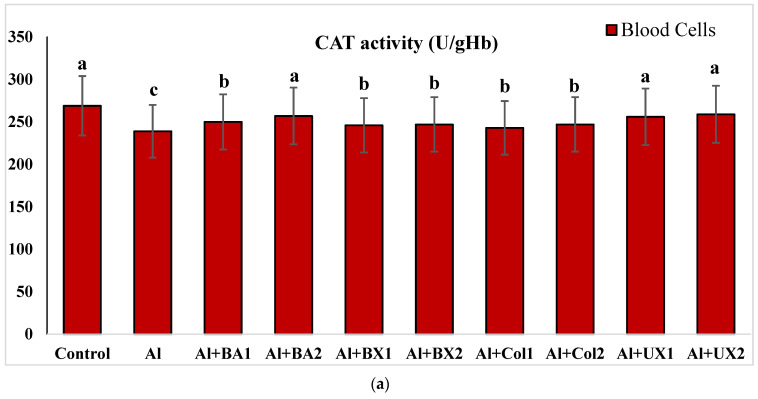

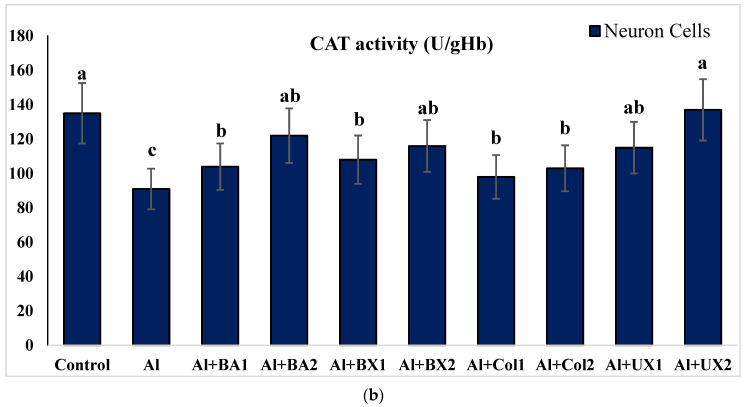
The effect of Al and boron treatments in (**a**) human blood and (**b**) PRCNs on CAT activity. Abbreviations are as in Figure 2. (Each superscript letter (a, b, c) represents statistically similar groups).

**Figure 4:**
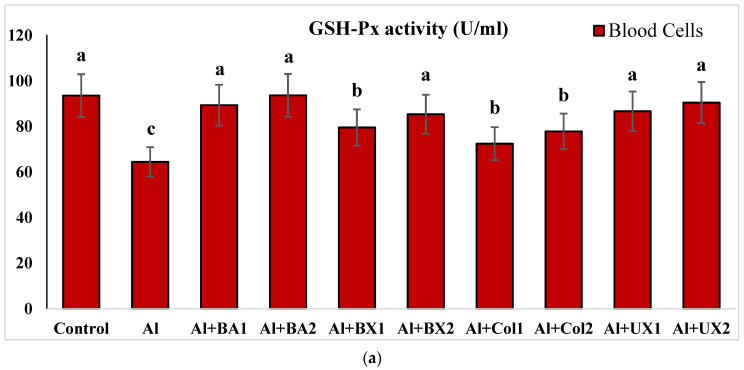

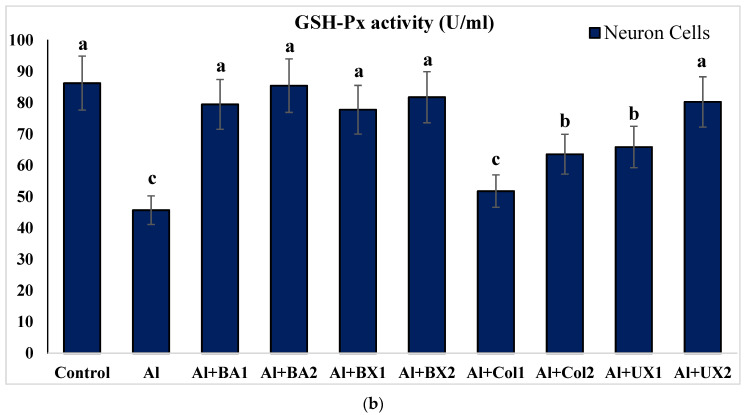
The effect of Al and boron treatments in (**a**) human blood and (**b**) PRCNs on GSH-Px activity. Abbreviations are as in Figure 2. (Each superscript letter (a, b, c) represents statistically similar groups).

**Figure 5:**
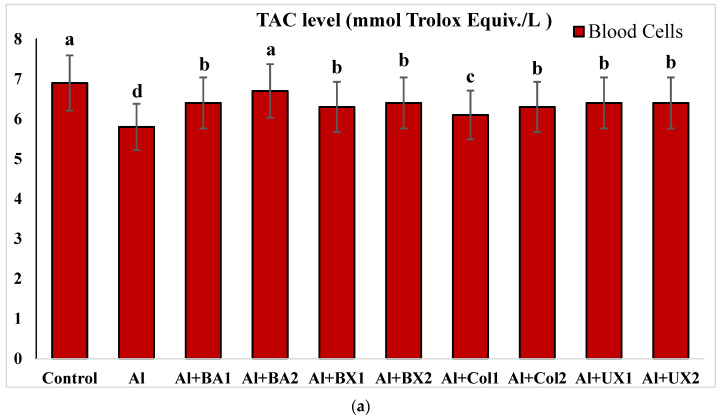

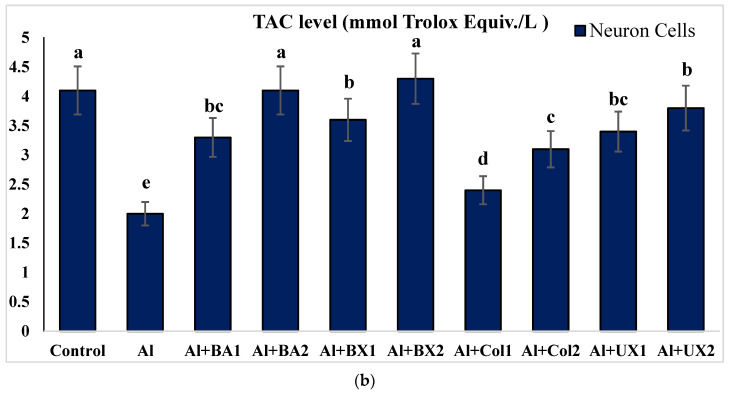
The effect of Al and boron treatments in (**a**) human blood and (**b**) PRCNs on TAC levels. Abbreviations are as in Figure 2. (Each superscript letter (a, b, c, d, e) represents statistically similar groups).

**Figure 6:**
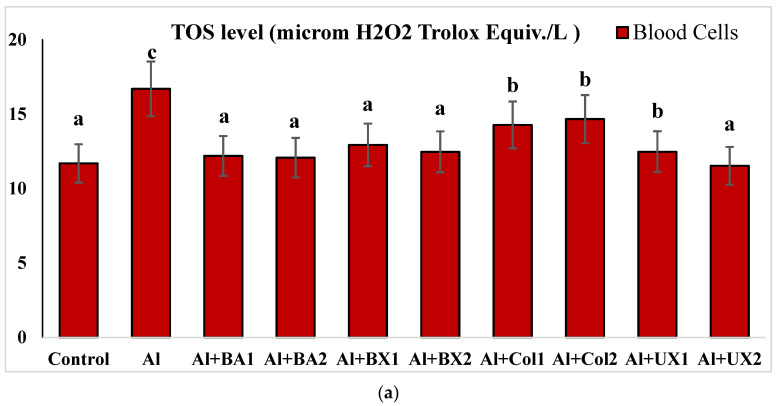

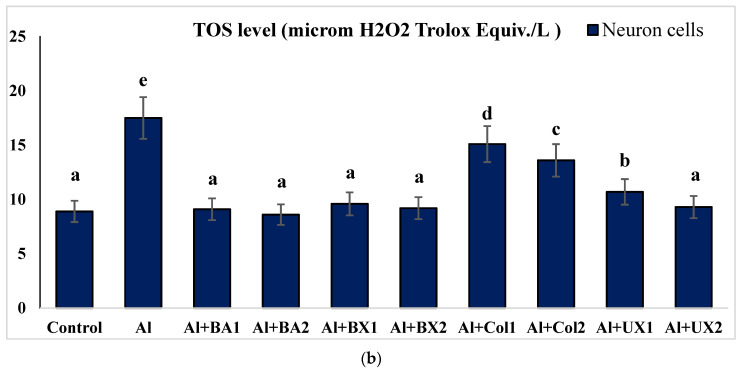
The effect of Al and boron treatments in (**a**) human blood and (**b**) PRCNs on TOS levels. Abbreviations are as in Figure 2. (Each superscript letter (a, b, c, d, e) represents statistically similar groups).

**Figure 7:**
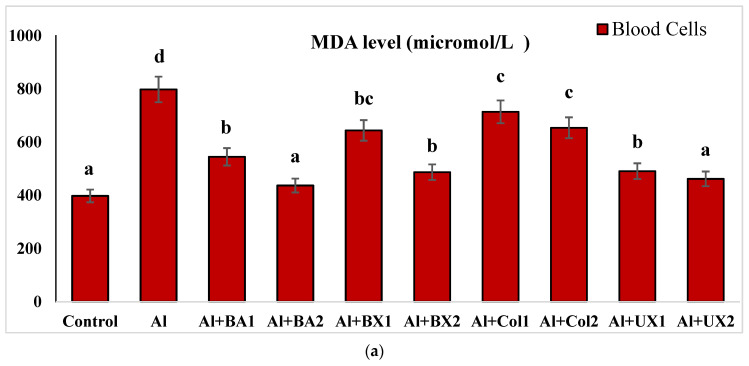

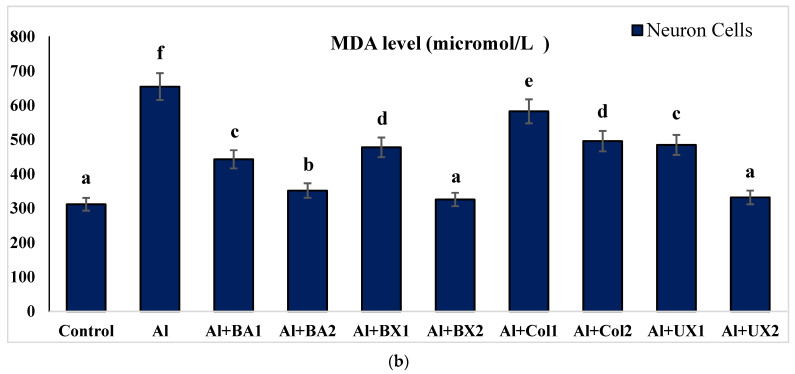
The effect of Al and boron treatments in (**a**) human blood and (**b**) PRCNs on MDA levels. Abbreviations are as in Figure 2. (Each superscript letter (a, b, c, d, e, f) represents statistically similar groups).

Human blood and PRCNs cultures were established, and Al and boron compounds were added to the culture medium at the determined concentrations (0.25, 0.5, 1, 2, 5, 10 and 20 ppm); then, the cultures were harvested after 72 h. Biochemical analyses including SOD, CAT, GSH-Px, MDA, TAC and TOS parameters were performed in peripheral human blood cultures. Our results revealed that the application of Al alone decreased SOD activity by 26.24% and 36.14%, 11.15% and 32.59% for CAT activity, 31.09% and 47.67% for GSH-Px activity and 16.18% and 50.72% for TAC levels in cultured human blood and PRCNs, respectively. Moreover, we observed that TOS levels increased by 42.73% and 96.63%, and MDA levels by 100.51% and 109.94% after Al exposure in cultured human blood and PRCNs cells, respectively. On the contrary, boron compounds improved in vitro antioxidant capacity depending on the type and applied concentration of the compound. In fact, BA (2, 5 and 10 ppm), BX (5 and 10 ppm), CO (10 ppm) and UX (5, 10 and 20 ppm) significantly increased (*p* < 0.05) SOD activity in both cell types. Similarly, BA (2, 5 and 10 ppm), BX (2, 5 and 10 ppm), CO (10 ppm) and UX (5 and 10 ppm) significantly increased CAT activity depending on the administrated dose. Similar to these changes in SOD and CAT activities, BA (10 and 20 ppm), BX (5 and 10 ppm), CO (10 ppm) and UX (2, 5 and 10 ppm) applications also positively affected GSH-Px enzyme activities of human blood and PRCNs cells. Concentrations of 5 and 10 ppm of BA, BX and UX and 5 and 10 ppm of CO increased the TAC levels of both cells compared to the control group. Considering the effects on antioxidant parameters, we observed that the compounds were generally beneficial at concentrations of 5 and 10 ppm, and BA, BX and UX compounds were superior to CO in terms of their effectiveness. On the other hand, the administered compounds did not alter TOS and MDA levels at all doses tested (0.25, 0.5, 1, 2, 5, 10 and 20 ppm). Moreover, co-application of boron compounds and Al ameliorated the oxidative effect by Al in both cell types, significantly increased the antioxidant enzyme activities, including SOD, CAT, and GSH-Px, supported TAC levels and minimized the levels of TOS and MDA contents in vitro. The results of the application of boron compounds (5 and 10 ppm) with Al (20 ppm) to the culture medium are summarized in Figure 2, Figure 3, Figure 4, Figure 5, Figure 6 and Figure 7.

The results of CA, MN and SCGE assays performed on peripheral human blood cultures are presented in Table 3. Our findings clearly demonstrate that all tested boron compounds were found to be non-genotoxic in vitro. Moreover, these compounds exhibited anti-genotoxicity properties against the genotoxic damage induced by Al depending on the type of boron compound. In addition, the effects of boron compounds on cell proliferation were assessed by calculating NDI values. The results of NDI analysis revealed that Al application alone significantly decreased the NDI values, but tested boron compounds alone did not suppress NDI values at both 5 and 10 ppm concentrations compared to untreated cell cultures. Moreover, the tested boron compounds were found to be protective at different doses against this suppressive effect by Al (Table 3).

**Figure 8:**
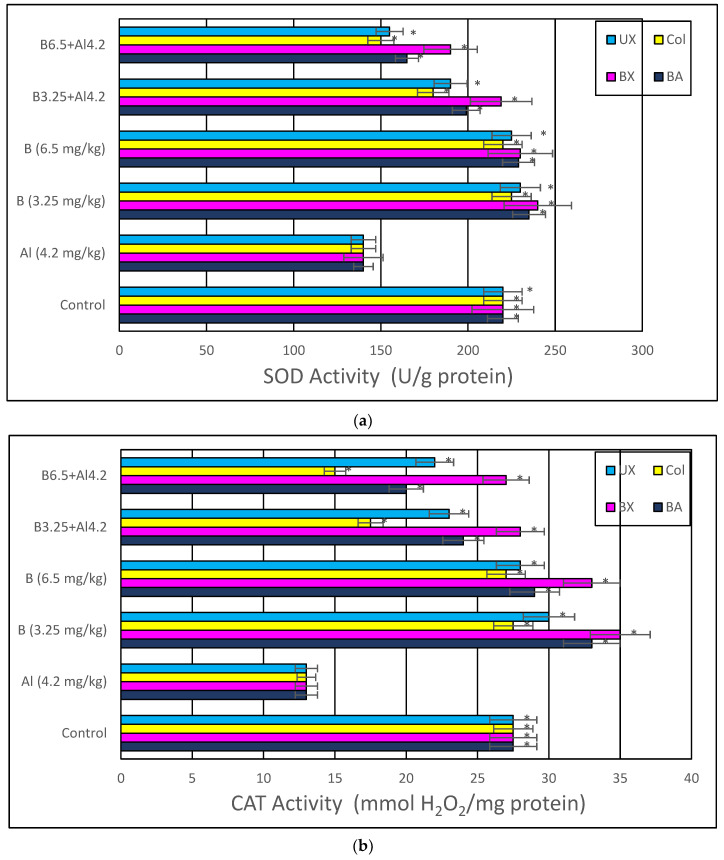

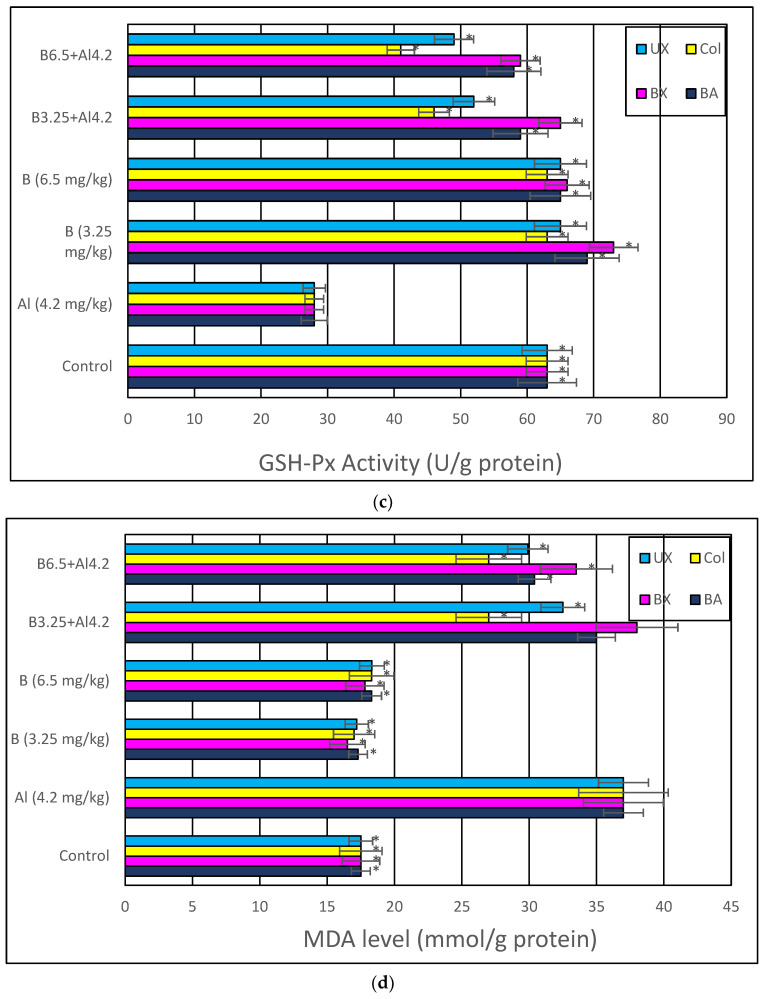

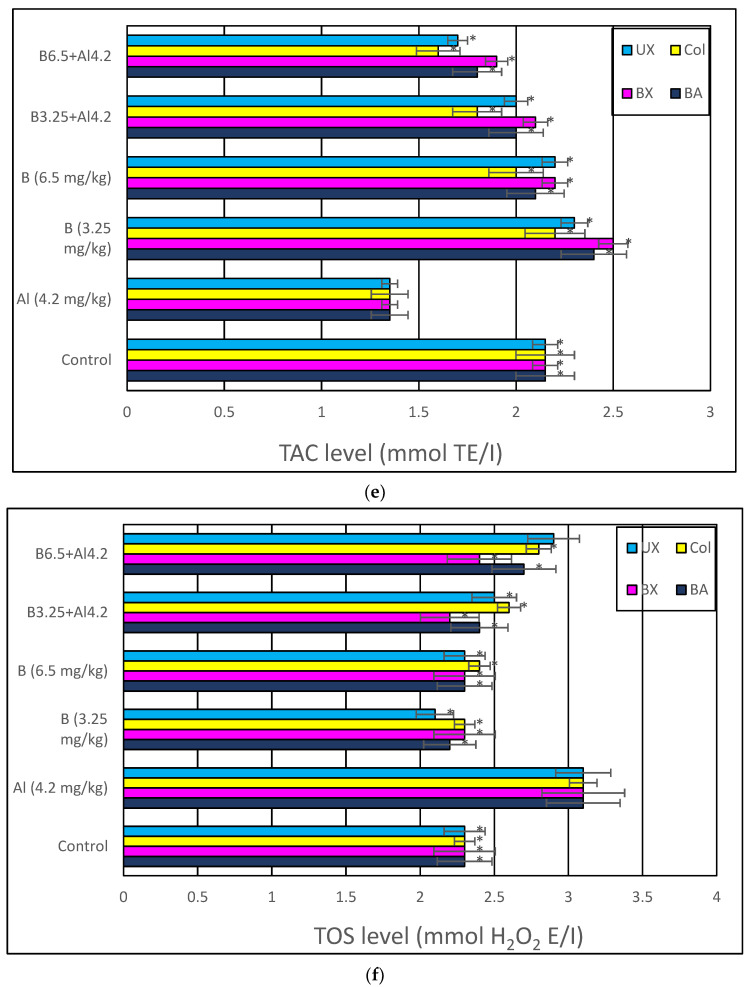
In vivo effects of treatments with boron compounds and Al on (**a**) SOD, (**b**) CAT and (**c**) GSH-Px enzyme activities, and (**d**) MDA, (**e**) TAC and (**f**) TOS levels in brain tissue. Symbol (*) represents a statically significant difference compared to the Al-treated group.

Treatment with Al (4.2 mg/kg b.w.) for 30 days triggered a distinct diminution in brain and serum activities of oxidative stress markers, including SOD, CAT, GSH-Px and TAC, while lipid peroxidation was remarkably elevated as monitored by MDA and TOS levels. The individual applications of each tested boron compound, including BA, BX, Col and UX supported antioxidant parameters without altering MDA and TOS levels. Additionally, the co-treatment with boron compounds at 3.26 and 6.5 mg/kg b.w. alleviated Al-induced alterations in oxidative biomarkers in both tissue types. The effectiveness of boron compounds in restoration of Al-induced oxidative damages in decreasing order was BX > BA > UX > Col (Figure 8 and Figure S1).

Table 4 reflects the rates of MNPKEs in rat bone marrow cells in different experimental groups. A significantly greater frequency of MNPKEs was observed in Al-treated rats compared to controls. Treatment with 4.2 mg/kg of Al resulted in an MNPKE frequency of 27.78‰, which was significantly (*p* < 0.05) higher than the 13.40‰ scored in the control animals. Conversely, the number of MNPKEs was not significantly different between the all-boron compound-treated rats at doses of 3.25 and 6.5 mg/kg to the control rats (*p* > 0.05).

The frequencies of CA are also shown in Table 4. The results of the CA assay revealed that Al-treatment induced about a seven-fold increase in total chromosomal aberrations rates compared to the control group. Likewise, exposure to Al showed an increase in total DNA damage as scored using SCGE assay when compared with control. The applications of BA, BX, UX and Col resulted in non-significant changes in the rates of both total chromosomal aberrations and damage scores as compared to that of control animals. Moreover, BA, BX, UX and Col treatment doses decreased Al-induced chromosome aberration rates and total damage scores. Our findings indicated that Al plus boron compound groups could protect from genotoxicity arising from Al (Table 4).

**Figure 9:**
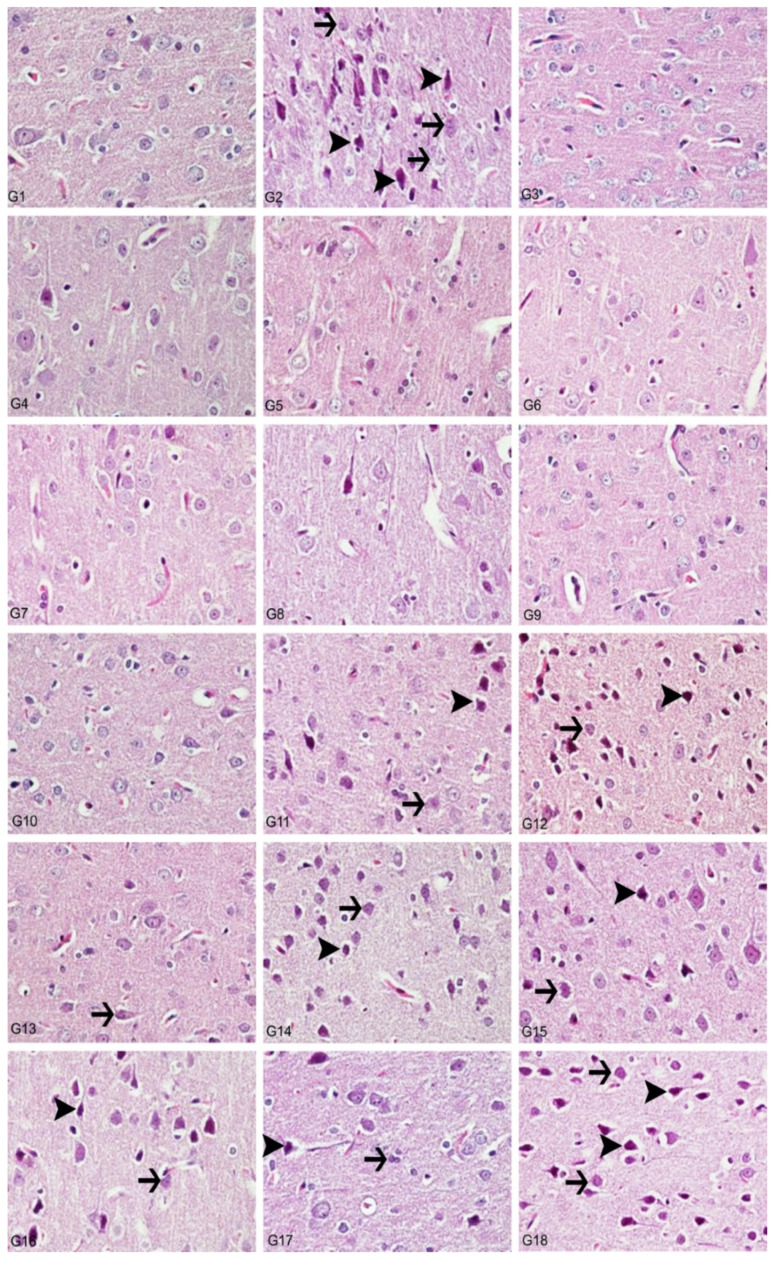
Cerebral cortex, degeneration of neurons (arrows), necrosis (arrowheads), hyperemia, H&E, 40X. (1) Control group, (2) Al-treated group for 30 days (as AlCl_3_, 4.2 mg/kg b.w.), (3) BA-treated group for 30 days (3.25 mg/kg b.w.), (4) BA-treated group for 30 days (6.5 mg/kg b.w.), (5) BX-treated group for 30 days (3.25 mg/kg b.w.), (6) BX-treated group for 30 days (6.5 mg/kg b.w.), (7) Col-treated group for 30 days (3.25 mg/kg b.w.), (8) Col-treated group for 30 days (6.5 mg/kg b.w.), (9) UX-treated group for 30 days (3.25 mg/kg b.w.), (10) UX-treated group for 30 days (6.5 mg/kg b.w.), (11) co-application of 3.25 mg/kg BA and 4.2 mg/kg Al, (12) co-application of 6.5 mg/kg BA and 4.2 mg/kg Al, (13) co-application of 3.25 mg/kg BX and 4.2 mg/kg Al, (14) co-application of 6.5 mg/kg BX and 4.2 mg/kg Al, (15) co-application of 3.25 mg/kg Col and 4.2 mg/kg Al, (16) co-application of 6.5 mg/kg Col and 4.2 mg/kg Al, (17) co-application of 3.25 mg/kg UX and 4.2 mg/kg Al, (18) co-application of 6.5 mg/kg UX and 4.2 mg/kg Al.

**Figure 10:**
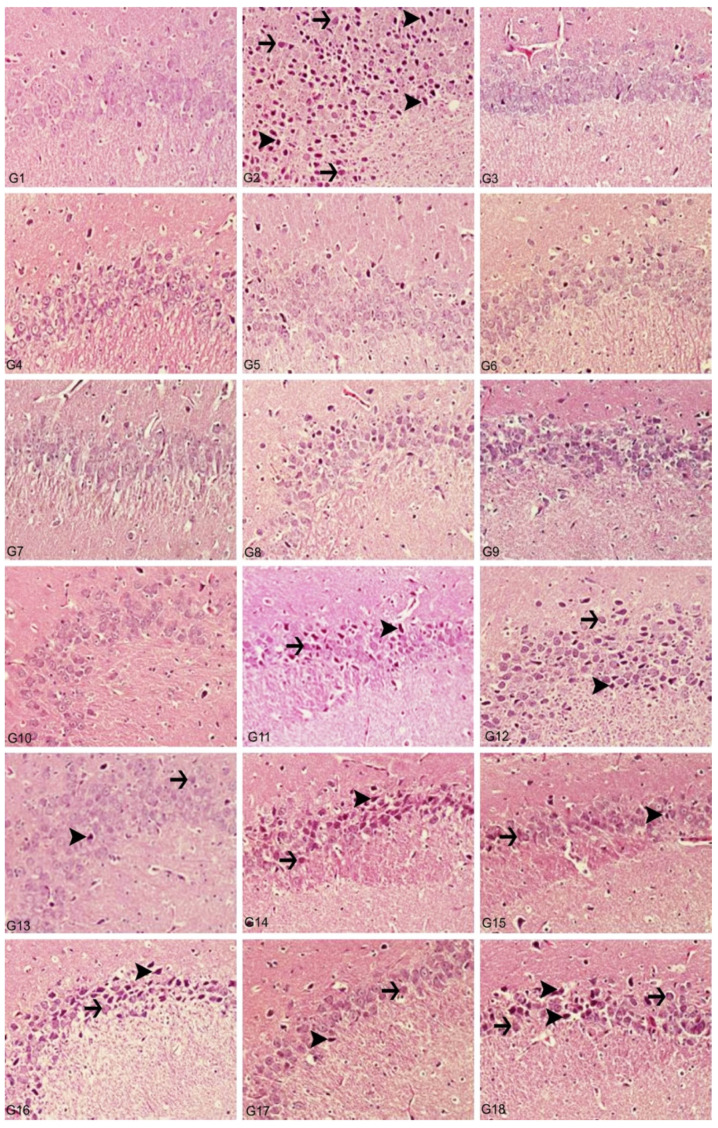
Hippocampus, degeneration in creatures (arrows), necrosis (arrowheads), hyperemia, H&E, 40X. (1) Control group, (2) Al-treated group for 30 days (as AlCl_3_, 4.2 mg/kg b.w.), (3) BA-treated group for 30 days (3.25 mg/kg b.w.), (4) BA-treated group for 30 days (6.5 mg/kg b.w.), (5) BX-treated group for 30 days (3.25 mg/kg b.w.), (6) BX-treated group for 30 days (6.5 mg/kg b.w.), (7) Col-treated group for 30 days (3.25 mg/kg b.w.), (8) Col-treated group for 30 days (6.5 mg/kg b.w.), (9) UX-treated group for 30 days (3.25 mg/kg b.w.), (10) UX-treated group for 30 days (6.5 mg/kg b.w.), (11) co-application of 3.25 mg/kg BA and 4.2 mg/kg Al, (12) co-application of 6.5 mg/kg BA and 4.2 mg/kg Al, (13) co-application of 3.25 mg/kg BX and 4.2 mg/kg Al, (14) co-application of 6.5 mg/kg BX and 4.2 mg/kg Al, (15) co-application of 3.25 mg/kg Col and 4.2 mg/kg Al, (16) co-application of 6.5 mg/kg Col and 4.2 mg/kg Al, (17) co-application of 3.25 mg/kg UX and 4.2 mg/kg Al, (18) co-application of 6.5 mg/kg UX and 4.2 mg/kg Al.

Histopathological analyzes were performed with light and electron microscopes on the preparations prepared from brain tissues taken from animals. Evaluations were made in the hippocampus and cortex regions of the rat brain. Compared to the control group, cytoplasmic swelling showed severe degeneration, chromatin condensation and pyknosis in the animal group given Al alone. In addition, we observed that the blood–brain barrier was severely impaired. Accordingly, edema findings were detected. Again, we observed that microglia, which are phagocytic cells, increased significantly. We observed that the light microscopic structure in the groups to which boron compounds were added alone was similar to the control groups. Moreover, we determined that the light microscopy structure in the low dose (3.25 mg/kg) of BA, BX, Col and UX groups given simultaneously with Al was similar to the structures in control, so that boron compounds protected hippocampal neurons from the neurotoxic effects of Al. Among all compounds, BX had the best protective effect. Although high dose (6.5 mg/kg) boron compounds were not very protective against Al, better microscopic structures were observed compared to the group in which aluminum was applied alone. When compared with Hematoxylin-eosin (H&E)-stained sections of cerebral cortices obtained from control group rats, histopathological findings were detected at a light microscopic level in tissue sections of the Al-treated group. Significant degenerative changes were also observed in hippocampal neurons. As an indicator of cellular damage, intense staining was remarkable in neurons, which acquired a pycnotic appearance due to cytoplasmic and nuclear shrinkage (Figure 9 and Figure 10).

In the histopathological examination of cerebral cortex tissues, we determined that the brain tissue samples examined in the control group and the high and low dose groups containing only boron compounds (BA, BX, Col, UX) had normal histological appearance (Figure 9). In the second group, the Al group, we observed severe degeneration in brain tissues and neurons, hyperchromasia and pycnosis in nuclei, and severe hyperemia in vessels (Figure 10). While mild degeneration and necrosis were observed in the low doses of the BA, Col and UX groups in the treatment groups, we found very mild degeneration in the low dose (3.25 mg/kg b.w. for 30 days) application of BX treatment, but no necrotic neurons (Figure 9). A statistically significant difference (*p* ˂ 0.05) was found when all groups of low-dose therapies were compared with the Al-treated group. Moderate degeneration, necrosis and hyperemia were observed in the brain tissues at high doses of the treatment groups (BA, BX, Col and UX) (Figure 10). However, this difference was not found to be statistically significant. Histopathological findings are summarized in Table 5.

In the histopathological examination of the hippocampus region in rat brain tissues, we observed that the hippocampus tissue had a normal histological structure in the groups in which control and boron compounds were administered alone (BA, BX, Col, UX) (Figure 10). In the examination of the hippocampus of the rats in the Al-treated group, we observed very severe degeneration in neurons in the granular layer, very severe necrotic cells with pycnotic nuclei and eosinophilic staining in the cytoplasm (Figure 10). In the histopathological examination of the low-dose BA, Col and UX groups applied in addition to the Al, we detected degeneration and necrosis in the cells in the moderate granular layer (Figure 10). In the BX low dose group, we found mild degeneration and necrosis in the hippocampus and granular layer cells (Figure 10). When these groups were compared with the Al-treated group, we found a statistically significant difference (*p* ˂ 0.05). At high doses of co-applications with boron compounds and Al, we observed severe degeneration and necrosis in the cells of the granular layer and hyperemia in the vessels in the hippocampus tissues (Figure 10). When these high-dose co-applications were compared with the Al-treated group, we determined that the difference between them was not statistically significant. Histopathological findings are summarized in Table 6.

**Figure 11:**
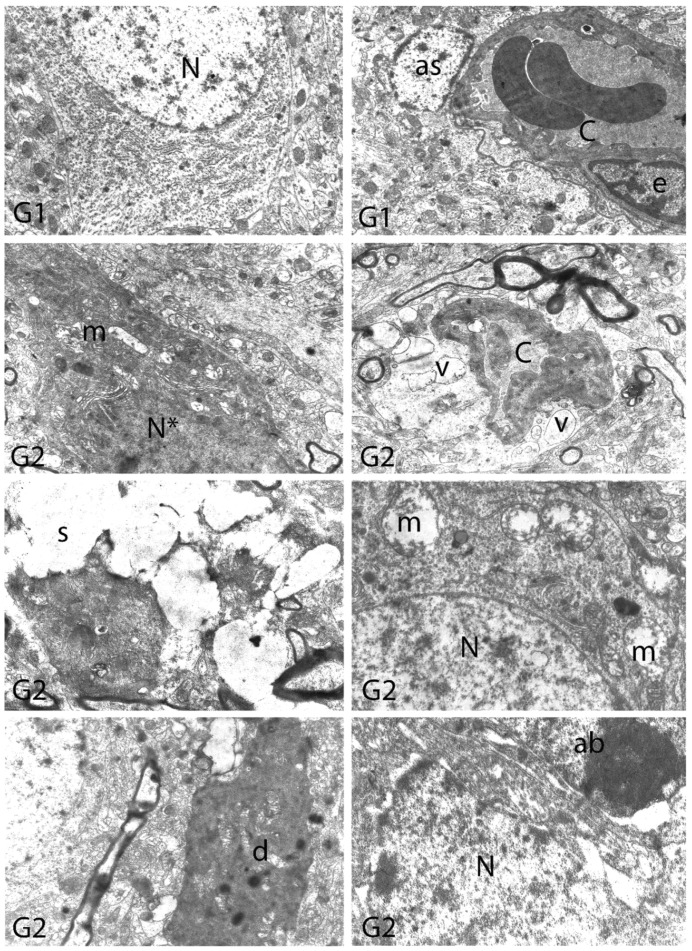
Electron micrographs of rat cerebral cortices from Groups 1 (G1) and 2 (G2). N, nucleus; C, capillary; e, endothelial cell nucleus; as, astrocyte nucleus; N*, pyknotic nucleus of a shrunken electron-dense neuron; m, swollen mitochondria; v, perivascular vacuolization and edema; s, swollen cytoplasm; d, neuronal debris; ab, apoptotic body. Group definitions are as in Figure 9.

**Figure 12:**
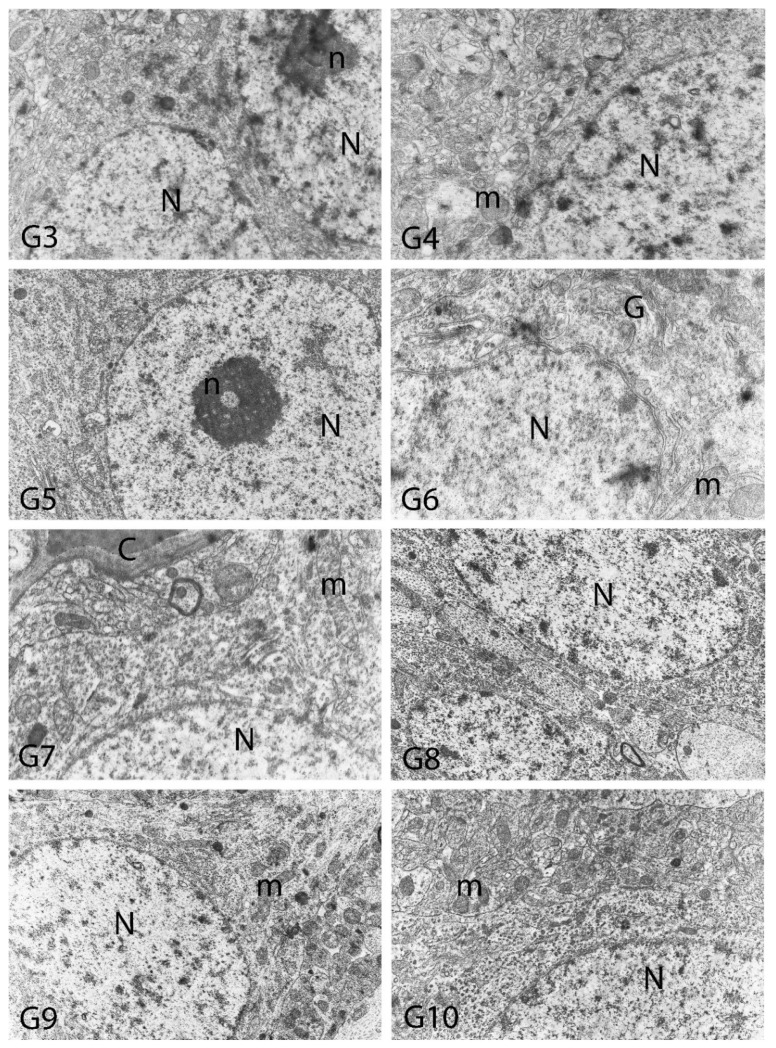
Electron micrographs of rat cerebral cortices from Groups 3 to 10 (G3–G10). N, nucleus; n, nucleolus; C, capillary; m, mitochondria; G, Golgi apparatus. Group definitions are as in Figure 9.

**Figure 13:**
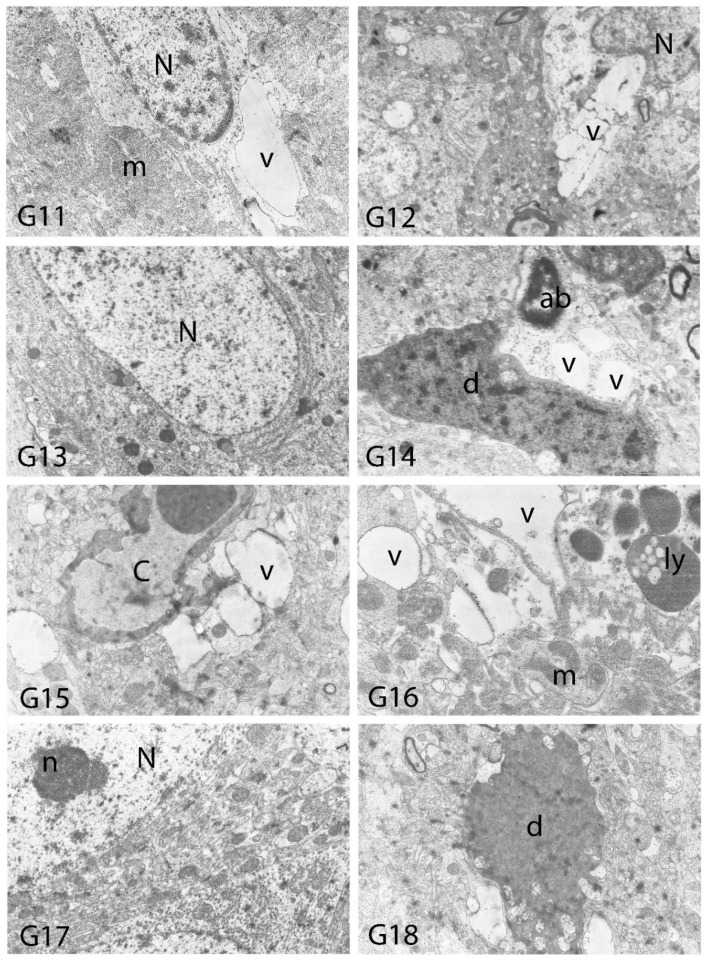
Electron micrographs of rat cerebral cortices from Groups 11 to 18 (G11–G18). N, nucleus; n, nucleolus; C, capillary; ab, apoptotic body m, mitochondria; v, vacuolar degeneration; ly, secondary lysosomes; d, neuronal debris. Group definitions are as in Figure 9.

**Figure 14:**
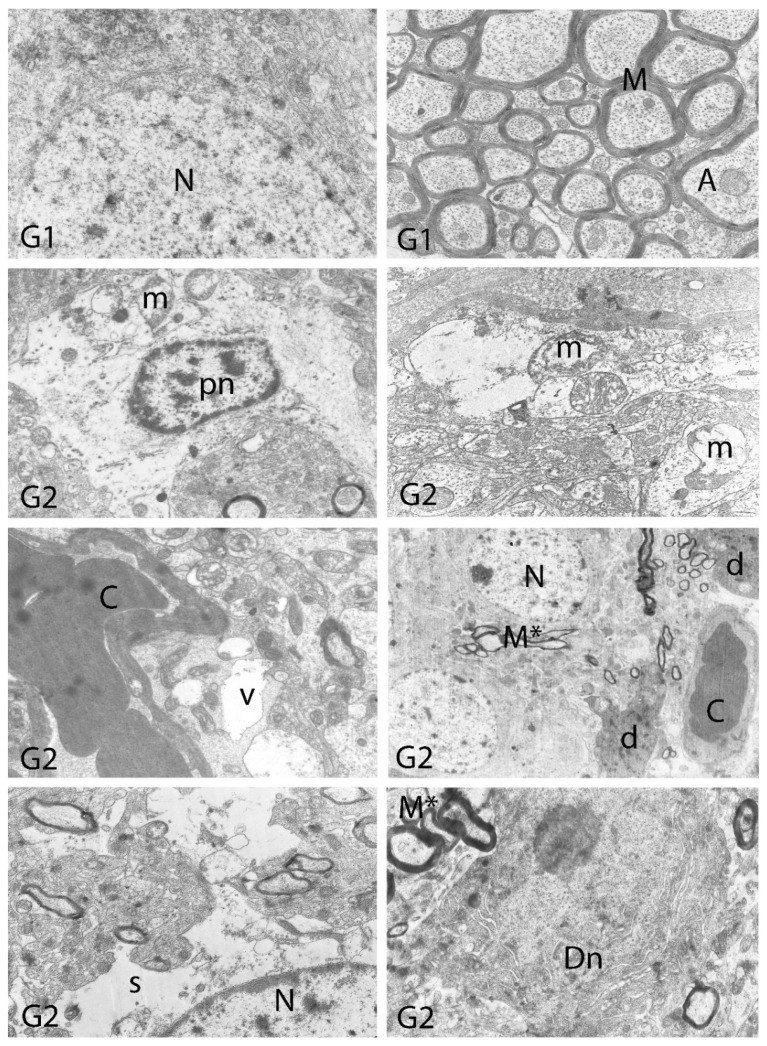
Electron micrographs of rat hippocampi from Groups 1 (G1) and 2 (G2). N, nucleus; M, intact myelin; A, axon; pn, pyknotic nucleus; m, damaged mitochondria; C, capillary; v, vacuolar degeneration; M*, distorted myelin figures; d, neuronal debris; s, swollen cytoplasm; Dn, electron dense degenerated neuron. Group definitions are as in Figure 9.

**Figure 15:**
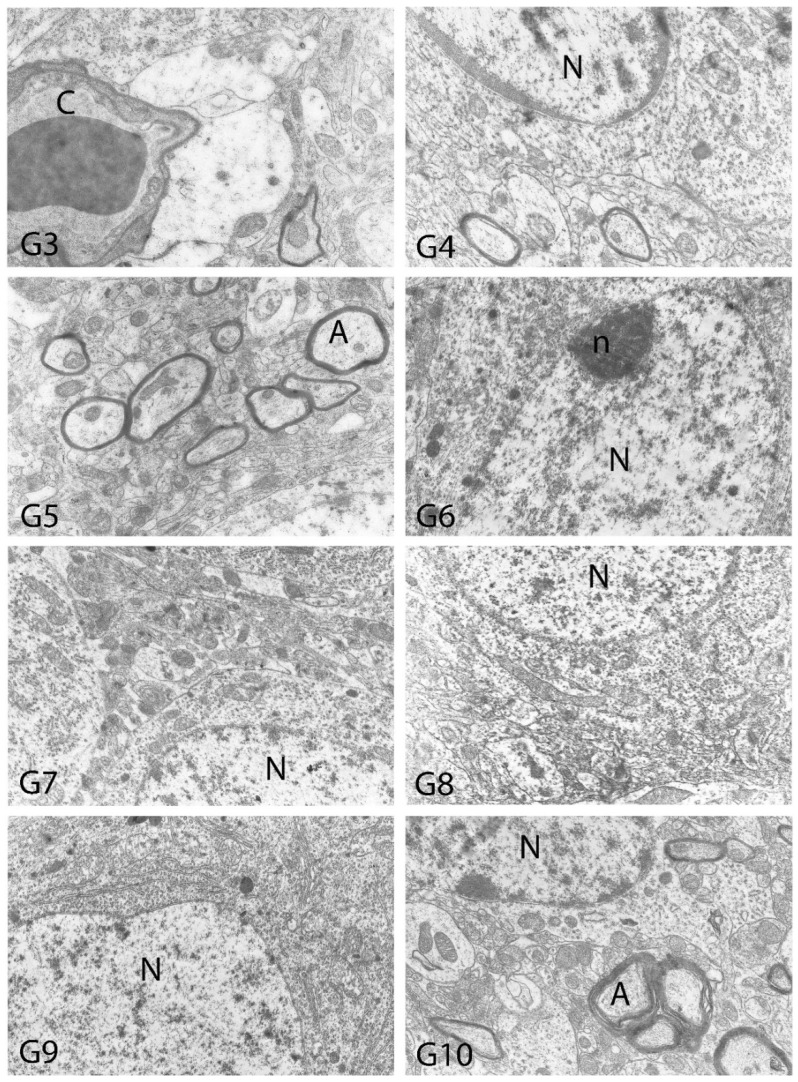
Electron micrographs of rat hippocampi from Groups 3 to 10 (G3–G10). N, nucleus; n, nucleolus; A, axon; C, capillary. Group definitions are as in Figure 9.

**Figure 16:**
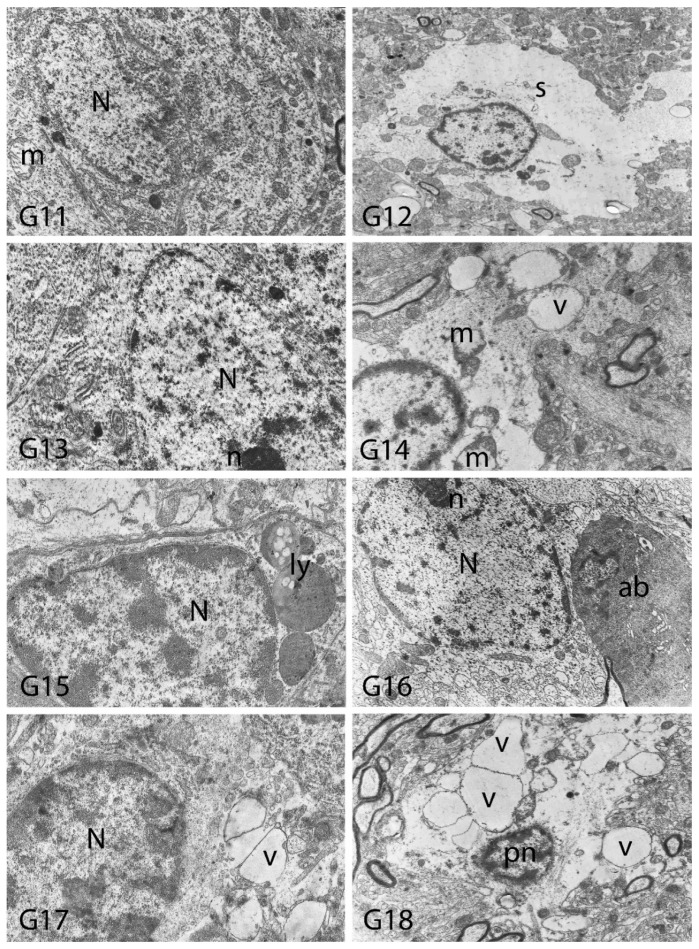
Electron micrographs of rat hippocampi from Groups 11 to 18 (G11–G18). N, nucleus; n, nucleolus; pn, pyknotic nucleus; m, damaged mitochondria; s, swollen cytoplasm; ly, secondary lysosomes; v, vacuolar degeneration; ab, apoptotic body. Group definitions are as in Figure 9.

We carried out electron microscopic observations to make a fine structural evaluation at the organelle level supported by the light microscopic data. In this context, we analyzed; the control group and other groups in which only boron compounds were applied (BA, BX, Col, UX). We observed that neurons of cerebral cortex and hippocampus samples had a large nucleus with a euchromatic appearance, loose chromatin structure, abundant granular endoplasmic reticulum cisterns and polyribosomes, and a few well-developed and close-localized nuclei. We also found Golgi apparatus, multiple mitochondria cut transversely or longitudinally, sporadic lysosomes. Vascular structures and myelin formation were evaluated as similar in the control group sections (Figure 11, Figure 12, Figure 13 and Figure 14). In electron microscopic sections of the Al-treated group, we observed vacuolar degeneration, perinuclear cytoplasmic swelling, chromatin condensation and nuclear pyknosis, mitochondrial damage, and numerous secondary lysosomes in both cortical and hippocampal neurons (Figure 11, Figure 12, Figure 13 and Figure 14). In this group, Golgi organelles and swollen and distorted mitochondria losing their cristae were seen (Figure 11, Figure 12, Figure 13 and Figure 14). In cortical and hippocampal sections, apoptotic neurons and apoptotic bodies containing their fragments were found, as well as electron-dense necrotic neurons. Nuclear fragments were observed in some apoptotic bodies, displaying the typical apoptotic nucleus appearance, with chromatin condensed to the periphery and stacked in a crescent shape just below the nuclear membrane (Figure 11, Figure 12 and Figure 13). In the treatment groups, we determined that the damage caused by Al in the cerebral cortex and hippocampus in low-dose boron compounds treatments with Al was significantly reduced, and findings such as vacuolar degeneration, perinuclear cytoplasmic swelling, chromatin condensation and nuclear pyknosis, mitochondrial damage were very mild (Figure 12, Figure 13, Figure 15 and Figure 16). We found that vacuolar degeneration in both cortical and hippocampal neurons, perinuclear cytoplasmic swelling, chromatin condensation and nuclear pyknosis, mitochondrial damage, numerous secondary lysosomes and apoptotic bodies in the Al-treated group were at a similar level in these groups as well (Figure 11, Figure 12, Figure 13, Figure 14, Figure 15 and Figure 16). Electron microscopic findings were observed to be compatible with histopathological findings. Electron microscopic findings are summarized in Table 7.

## 4. Discussion

Previous in vivo and in vitro studies clearly revealed that aluminum (Al) exposure caused the formation of amyloid plaques, which initiate the pathogenesis of AD. Therefore, chronic Al exposure, as in transgenic mouse models, is frequently used as an experimental AD model [52,53,54,55]. The discovery of effective preservatives against Al is one of the most popular topics of recent times, as Al has very strong neurotoxic effects [53,56]. The present research demonstrated for the first time that treatment with boron compounds (BA, BX, Col and UX) had positive results against the toxicity of Al on brain and blood tissues based on different biochemical, genetic and histological analyses.

It has been reported that boron has a positive role on brain and cognitive function in humans [57]. However, no histological, genetic or biochemical research has been found in the literature to explain its physiological role. With this study, it has been shown that boron compounds have a protective role against Al exposure via the prevention of neuronal loss. With respect to our in vivo studies, we obtained light and electron microscopic data similar to the control group in the brain cortex and hippocampus of the BA, BX, Col and UX groups. We also determined that boron compounds (especially BA, BX and UX) applied in low doses (3.25 mg/kg) reduced the harmful effects of Al application at different levels. Accordingly, it is thought that boron may have a remarkable protective effect against Al in mitochondria of neurons. Since oxidative stress develops when levels of antioxidants fall, the activities of cellular antioxidant enzymes have been seen to be particularly important in cell defense [58,59]. Although the relationship between Al toxicity versus boron and iron metabolism is not known, one of the valid hypotheses is that boron may affect oxidative metabolism by strengthening the antioxidant defense system in animals [60]. As a matter of fact, the application of appropriate amounts of boron provided protection for the lungs from forming reactive oxygen species (ROS) and exerted beneficial effects on inflammation [61]. Again, the relationship between metabolic functioning in humans and animals with the physiological amounts of boron in the diet (as BA and BX) has been determined with the protective role of BA and BX against liver necrosis in different degrees, as well as the positive effects of boron on normal liver metabolism [62]. A recent study showed that different boron compounds increased the antioxidant enzyme activities with low levels of supplements without causing oxidative stress on blood cells [17]. On the contrary, a significant decrease in the antioxidant enzyme activities of the neuron and glia cells of the rat brain was detected due to Al exposure [63]. Furthermore, the promotion of oxidative stress increased in conjunction with the direct effect of Al on enzyme molecules or their synthesis. Thus, some neurodegenerative disorders may develop due to decreased antioxidant activity [64]. Boron can act as an indirect proton donor, which has a unique effect on cell membrane structure and function [65]. Thus, it has been noted that cyclic adenosine mono phosphates (cAMP), whose concentration increases with the effect of boron, can interfere with the oxidative phosphorylation metabolism in mitochondria and inhibit hydrolytic enzyme activities [66]. At this point, the antioxidative features of examined boron compounds such as BA, BX, Col and UX on neuronal cells might be associated with their critical roles in mitochondrial dynamics.

On the other hand, the increase in the content of zinc in the tissue accelerated the formation of free radicals and the shaping of senile plaques [67,68]. It has been stated that Al can significantly increase the amount of zinc (Zn) in the brain, causing neuronal damage observed in AD [69]. Moreover, Zn accumulated in cells also had a direct effect on mitochondria and caused the apoptosis of these cells [70]. However, it has been noted that pathogenicity can be prevented by the injection of a Zn-holding agent, since the accumulation of metal occurs before neurodegeneration [70]. It is thought that a similar effect may occur in the brain due to supplementation with boron in order to prevent the loss of neuron against Al in this study.

It has been reported that aluminum can affect synaptic plasma membranes, microsomes and phospholipid composition of the myelin sheath, as well as the kinetic properties of acetylcholinesterase enzyme (AChE) in the brain [71]. It has been reported that Al could affect delta-aminolevulinic acid dehydratase (ALA-D) activity in the liver, kidney and brain, not only in vitro conditions, but also in vivo conditions [5]. It is known that the ALA-D enzyme also promotes lipid peroxidation in the brain cortex and cerebellum and can cause oxidative damage to cellular structures and macromolecules with increased ROS species [72]. However, boron in the diet was able to affect behavior and brain composition in rats through the cell membrane [73]. A previous study showed that in human fetal tissue, boron inhibits ALA-D activity by complexing with hydroxyl groups [74]. It has been stated that the boron can easily pass through the lipid layer of the cell membranes either directly by passive diffusion or through the channels in the membrane [75]. It has also been reported that BA can regulate membrane permeability by modulation of the activities of enzymes that cause oxidation in liver cells and lipid peroxidation [62]. In the present study, boron applications did not cause any degeneration in the myelin sheaths of nerve cells in the brain; on the contrary, they provided protection against Al toxicity by providing a similar membrane structure as in control.

In the present study, it was shown that the genetic damages in the Al-treated groups treated simultaneously with boron compounds observed by SCGE, CA and MN assays were lower than the solely Al-treated group. This finding revealed that the tested boron compounds have antigenotoxic effects against AlCl_3_ in vivo and in vitro conditions. It has been reported by the World Health Organization that boron exposure cannot be associated with genotoxicity in humans and animals [76]. Similarly, many in vitro and in vivo mutagenicity tests (especially on BA and BX) have shown that boron compounds are not genotoxic [15,33,77]. Furthermore, previous findings have found that boron compounds have antigenotoxic effects against various toxic materials such as titanium, thioacetamide and aflatoxins [78,79].

Various researchers have reported that metal compounds have toxic effects on peripheral red and white blood cells [80,81,82]. It has been reported that oxidative stress is highly effective in the onset of metal toxicity in cells [83,84]. The present study has shown that oxidative stress increases in erythrocytes due to the MDA level after exposure to Al, and this may be related to increases in MN, CA and comet frequencies in cells. Increased MDA level due to treatments with metals has been accepted as an essential indicator of increased lipid peroxidation and oxidative state in many lipid systems such as plasma, organ and cell membranes [85,86]. Therefore, gene expressions and cell proliferation could be affected [87]. Free radicals occurring in the presence of metals can cause modifications in DNA bases, acceleration of lipid peroxidation and changes in calcium and sulfhydryl homeostasis [88,89,90]. It is known that the hydroxyl radical reacts with all components of the DNA molecule and causes damage to the deoxyribose skeleton with both purine and pyrimidine bases. Moreover, it has been reported that these permanent changes in genetic material as a result of oxidative stress are the first steps in mutagenesis, carcinogenesis and aging [91,92]. The MN method has made it possible to measure both chromosome breaks and chromosome loss and non-separation in clastogenic and aneugenic situations [93]. On the other hand, CA has been evaluated as an important cytogenetic parameter in assessing the reliability of chemicals in terms of mutagenicity [94]. Their high sensitivity in detecting different levels of DNA damage and applicability even at the single cell level are the main reasons why the comet or SCGE methods are preferred in genotoxicity studies [95]. Within the scope of the study, the CA/cell, MN/1000 cell and total damage score values observed after Al exposure were significantly higher than the control group values (*p* < 0.05), as a result of genetic studies performed under in vitro (human lymphocyte cultures) and in vivo (rat bone marrow) conditions. This finding is in line with previous reports recorded in the literature. As a matter of fact, it has been noted that Al compounds have a very high affinity for DNA and RNA and that they can form complexes with DNA [96,97]. The presence of genotoxic effects of various aluminum compounds, mainly aluminum chloride and aluminum sulphate, has been demonstrated in mammalian cells (by MN, CA and sister chromatid exchange tests (SCE)) and bacteria (salmonella/AMES test) [98,99,100].

Neurotoxicity due to Al exposure has been demonstrated by both clinical observations and animal studies [101,102,103,104,105,106]. There are two reasons for the selection of the cerebral cortex and hippocampus regions in this study: the first is that Al affects the cerebral cortex and hippocampus much more seriously than any part of the central nervous system, and the second is that this metal has been found to have toxic effects on the pyramidal neurons in the cortex in vitro conditions [107,108,109]. In the current study, cytoplasmic swelling in neurons, chromatin condensation and pyknosis, as well as vacuolar degeneration, perinuclear cytoplasmic swelling, chromatin condensation and nuclear pyknosis, mitochondrial damage, multiple secondary lysosomes, disruption in the integrity of the myelin layer, and changes in the myelin lamellas were noted due to Al exposure in rats. These histopathological findings were also in line with the results of previous researchers [110,111,112,113]. Electron microscopic observations revealed that the main targets of AI in cells are myelin and mitochondria. In the spinal cord, Al has been reported to damage the myelin sheaths. Al affected mitochondria morphology and its function [114,115,116]. Various studies have shown that aluminum causes tissue damage by increasing pro-oxidative and inflammation mechanisms [117,118,119]. Within the scope of the project, in vivo and in vitro biochemical studies (with parameters SOD, CAT, GSH-Px, MDA, TAK and TOD) on brain and blood have clearly revealed that Al exposure significantly creates oxidative stress. Supporting our findings, a recent study reported that more pronounced oxidative damage was observed in the brain and liver than in the bone and kidney following exposure to Al [120]. Again, parallel to our findings, it has been noted that in vivo and in vitro exposure to Al causes significant damage to blood tissue through oxidative stress [26,121,122]. Moreover, it has been reported that due to oxidative stress, changes have been observed in the morphology and fine structure of mitochondria [123]. At this point, one reason for Al-induced oxidative damage has been explained by the fact that this metal can increase the amount of free and reduced iron in cells by altering iron metabolism [124]. Studies have shown that increased iron catalyzes the formation of superoxide anions leads to changes in the mitochondrial dynamics. This mechanism has been accepted as one of the most important causes of neuron loss [125,126,127,128]. However, whether absorbed Al causes neuron degeneration is a matter of debate. The findings of the project revealed that AI caused significant (*p* < 0.05) degrees of neuron degeneration in neuron cultures of the brain cortex and hippocampus compared to controls. Thus, this study seems to support the results of the researchers who reported that severe pathological conditions such as neuron loss in rat brain tissues were caused by Al exposure [129,130,131,132].

## 5. Conclusions

In conclusion, we determined the protective role of boron compounds (especially BA, BX and UX) against the pathological effects of chronic Al in the brain and blood for the first time. In addition to our in vitro findings, we also reported the absence of in vivo genotoxic/anti-genotoxic effects of boron compounds on experimental animals for the first time. In the light of the findings, we propose that low doses (3.25 mg/kg) of boron administration can prevent the emergence of various neurodegenerative and hematological diseases. Moreover, non-cytotoxic and non-genotoxic effects at these concentrations make boron compounds safe to use for cytoprotective purposes. The findings from our study highlighted that the administration with boron could suppress oxidative stress, reduce genotoxic damage and ameliorate the structural and ultrastructural architecture of the brain. As there are limited therapeutic options for neurodegenerative disorders, boron supplementation may provide an effective and safe, as well as economic, therapeutic option in the management of these disorders. Thus, our research results will contribute significantly to studies on pharmacological evaluation of BX, UX, Col and BA, which have just started to be used for medical, pharmaceutical and nutritional purposes.

## Figures and Tables

**Table 1:** The cytotoxicity of boron compounds and aluminum in comparison to untreated cultures.

Treatments (as ppm)	*p*							
	Blood Cells				PRCNs			
	BA	BX	Col	UX	BA	BX	Col	UX
0.01	1.00	0.97	0.98	1.00	1.00	1.00	0.97	1.00
0.02	1.00	0.95	0.96	1.00	1.00	1.00	0.93	1.00
0.05	1.00	0.96	0.94	1.00	0.96	1.00	0.90	0.97
0.1	0.97	0.95	0.96	0.98	0.93	0.96	0.86	0.90
0.2	0.95	0.92	0.91	0.96	0.90	0.94	0.90	0.86
0.5	0.94	0.90	0.84	0.94	0.85	0.90	0.72	0.79
1	0.94	0.85	0.81	0.95	0.82	0.86	0.78	0.77
2	0.90	0.84	0.79	0.92	0.74	0.88	0.68	0.84
5	0.86	0.81	0.76	0.87	0.72	0.82	0.66	0.70
10	0.85	0.79	0.73	0.85	0.78	0.77	0.61	0.66
20	0.83	0.74	0.69	0.83	0.71	0.75	0.53	0.67
AlCl_3_	0.00 *				0.00 *			

Symbol (*) represents a statistically significant decrease in cell viability compared to the control group (untreated cultures) and *p* < 0.05 was accepted as significant.

**Table 2:** The cytotoxicity of co-applications with boron compounds and Al in comparison to only Al-treated cultures.

Treatments (as ppm)	*p*							
	Blood Cells				PRCNs			
	BA	BX	Col	UX	BA	BX	Col	UX
0.01	0.87	0.94	0.96	0.92	0.91	0.82	0.93	0.86
0.02	0.79	0.86	0.91	0.83	0.88	0.87	0.90	0.71
0.05	0.76	0.72	0.87	0.65	0.79	0.74	0.83	0.74
0.1	0.67	0.55	0.79	0.62	0.66	0.72	0.78	0.67
0.2	0.59	0.49 *	0.72	0.54	0.70	0.65	0.74	0.63
0.5	0.04 *	0.06 *	0.65	0.04 *	0.56	0.53	0.60	0.56
1	0.04 *	0.33 *	0.59	0.04 *	0.51	0.02 *	0.53	0.04 *
2	0.04 *	0.21 *	0.53	0.03 *	0.04 *	0.02 *	0.04 *	0.04 *
5	0.03 *	0.19 *	0.45 *	0.02 *	0.04 *	0.02 *	0.04 *	0.02 *
10	0.02 *	0.02 *	0.04 *	0.02 *	0.03 *	0.02 *	0.04 *	0.03 *
20	0.01 *	0.06 *	0.03	0.01 *	0.07 *	0.02 *	0.54	0.03 *
Control	0.00 *				0.00 *			

Symbol (*) represents a statistically significant increase in cell viability compared to only Al-treated cultures and *p* < 0.05 was accepted as significant.

**Table 3:** The genotoxicity of boron compounds and Al on the CA, MN, SCGE and NDI rates in peripheric human blood cells.

Groups	MN/1000 Cell	CA/Cell	Total Damage Score	NDI (%)
Control	3.82 ± 0.26	0.18 ± 0.02	32.25 ± 4.76	1.22 ± 0.05
Al (20 mg/L)	14.21 ± 1.55 *	1.14 ± 0.09 *	167.75 ± 14.65 *	1.09 ± 0.03 *
BA1 (5 mg/L)	2.72 ± 0.14	0.18 ± 0.02	34.00 ± 3.65	1.20 ± 0.02
BA2 (10 mg/L)	2.85 ± 0.26	0.16 ± 0.01	30.75 ± 3.35	1.18 ± 0.03
BX1 (5 mg/L)	3.76 ± 0.44	0.20 ± 0.03	33.25 ± 3.48	1.24 ± 0.04
BX2 10 mg/L)	3.68 ± 0.29	0.22 ± 0.02	34.50 ± 2.65	1.22 ± 0.03
Col1 (5 mg/L)	3.91 ± 0.33	0.18 ± 0.02	34.75 ± 4.15	1.20 ± 0.03
Col2 10 mg/L)	4.01 ± 0.37	0.21 ± 0.03	36.50 ± 3.70	1.19 ± 0.02
UX1 (5 mg/L)	3.94 ± 0.41	0.17 ± 0.02	30.25 ± 3.45	1.24 ± 0.03
UX2 10 mg/L)	3.56 ± 0.34	0.18 ± 0.03	35.50 ± 4.20	1.21 ± 0.03
BA1 + Al	6.42 ± 0.48 *	0.44 ± 0.02 *	79.50 ± 6.80 *	1.15 ± 0.02
BA2 + Al	5.74 ± 0.45 *	0.48 ± 0.03 *	71.25 ± 6.22 *	1.17 ± 0.02
BX1 + Al	5.90 ± 0.32 *	0.64 ± 0.04 *	89.00 ± 9.38 *	1.13 ± 0.03 *
BX2 + Al	6.23 ± 0.38 *	0.57 ± 0.03 *	87.75 ± 9.04 *	1.16 ± 0.02
Col1 + Al	8.91 ± 0.61 *	0.81 ± 0.05 *	149.25 ± 12.66 *	1.12 ± 0.02 *
Col2 + Al	8.44 ± 0.55 *	0.75 ± 0.06 *	135.00 ± 15.42 *	1.12 ± 0.03 *
UX1 + Al	5.50 ± 0.35 *	0.57 ± 0.04 *	68.50 ± 7.20 *	1.16 ± 0.03
UX2 + Al	5.73 ± 0.46 *	0.47 ± 0.04 *	44.25 ± 4.60 *	1.18 ± 0.03

* symbol presents statistical difference from the control group at a level of *p* < 0.05. Control: untreated cells and positive control: Al application.

**Table 4:** The genotoxicity of boron compounds and Al on the CA, MN, SCGE and NDI rates in rat bone marrow cells.

Groups	MNPKE/1000 PKE	CA/Cell	Total Damage Score
Control	13.40 ± 1.18	0.66 ± 0.08	19.35 ± 2.44
Al (20 mg/L)	27.78 ± 1.86 *	4.58 ± 0.16 *	66.35 ± 7.98 *
BA1 (5 mg/L)	12.65 ± 0.96	0.54 ± 0.12	21.40 ± 1.74
BA2 (10 mg/L)	12.89 ± 1.32	0.52 ± 0.16	20.34 ± 2.55
BX1 (5 mg/L)	12.20 ± 0.89	0.58 ± 0.11	19.33 ± 1.80
BX2 10 mg/L)	12.93 ± 1.16	0.51 ± 0.18	20.08 ± 2.14
Col1 (5 mg/L)	12.84 ± 1.09	0.62 ± 0.21	20.80 ± 1.92
Col2 10 mg/L)	13.15 ± 1.33	0.65 ± 0.19	21.66 ± 2.15
UX1 (5 mg/L)	12.74 ± 0.92	0.54 ± 0.13	21.85 ± 1.92
UX2 10 m/L)	12.96 ± 0.78	0.61 ± 0.22	20.66 ± 2.38
BA1 + Al	17.04 ± 1.53 *	2.70 ± 0.28 *	30.72 ± 2.78 *
BA2 + Al	18.45 ± 1.22 *	3.16 ± 0.24 *	37.85 ± 3.25 *
BX1 + Al	16.70 ± 1.42 *	2.40 ± 0.19 *	27.20 ± 2.82 *
BX2 + Al	17.35 ± 1.30 *	2.67 ± 0.16 *	34.41 ± 3.16 *
Col1 + Al	19.86 ± 1.50 *	2.86 ± 0.22 *	42.36 ± 3.40 *
Col2 + Al	23.75 ± 1.72 *	3.68 ± 0.25 *	45.08 ± 4.13 *
UX1 + Al	17.92 ± 1.05 *	3.38 ± 0.26 *	34.23 ± 3.15 *
UX2 + Al	21.55 ± 1.77 *	4.23 ± 0.24 *	42.76 ± 4.07 *

* symbol presents statistical difference from the control group at a level of *p* < 0.05.

**Table 5:** Scoring of histopathological findings observed in the cerebral cortex after treatments with boron compounds and Al. (- nonsignificant; + significant at %0.01; ++ significant at %0.1; +++ significant at %1; ++++ significant at %5, +++++ significant at 10%).

	Degeneration in Neurons	Necrosis in Neurons	Hyperemia in Vessels
Control	-	-	-
Al (4.2 mg/kg)	++++	++++	++++
BA (3.25 mg/kg)	-	-	-
BA (6.50 mg/kg)	-	-	-
BX (3.25 mg/kg)	-	-	-
BX (6.50 mg/kg)	-	-	-
Col (3.25 mg/kg)	-	-	-
Col (6.50 mg/kg)	-	-	-
UX (3.25 mg/kg)	-	-	-
UX (6.50 mg/kg)	-	-	-
BA (3.25 mg/kg) + Al (4.2 mg/kg)	++	++	++
BA (6.50 mg/kg) + Al (4.2 mg/kg)	+++	+++	+++
BX (3.25 mg/kg) + Al (4.2 mg/kg)	+	-	+
BX (6.50 mg/kg) + Al (4.2 mg/kg)	++	++	+++
Col (3.25 mg/kg) + Al (4.2 mg/kg)	++	++	++
Col (6.50 mg/kg) + Al (4.2 mg/kg)	+++	+++	+++
UX (3.25 mg/kg) + Al (4.2 mg/kg)	+	++	++
UX (6.50 mg/kg + Al (4.2 mg/kg)	+++	+++	+++

**Table 6:** Scoring of histopathological findings observed in the hippocampus after treatments with boron compounds and Al. (- nonsignificant; + significant at %0.01; ++ significant at %0.1; +++ significant at %1; ++++ significant at %5, +++++ significant at 10%).

	Degeneration in Neurons	Necrosis in Neurons	Hyperemia in Vessels
Control	-	-	-
Al (4.2 mg/kg)	+++++	+++++	+++++
BA (3.25 mg/kg)	-	-	-
BA (6.50 mg/kg)	-	-	-
BX (3.25 mg/kg)	-	-	-
BX (6.50 mg/kg)	-	-	-
Col (3.25 mg/kg)	-	-	-
Col (6.50 mg/kg)	-	-	-
UX (3.25 mg/kg)	-	-	-
UX (6.50 mg/kg)	-	-	-
BA (3.25 mg/kg) + Al (4.2 mg/kg)	++	++	+++
BA (6.50 mg/kg) + Al (4.2 mg/kg)	++++	++++	++++
BX (3.25 mg/kg) + Al (4.2 mg/kg)	++	+	++
BX (6.50 mg/kg) + Al (4.2 mg/kg)	++++	++++	+++
Col (3.25 mg/kg) + Al (4.2 mg/kg)	+++	+++	+++
Col (6.50 mg/kg) + Al (4.2 mg/kg)	++++	++++	++++
UX (3.25 mg/kg) + Al (4.2 mg/kg)	+++	++	+++
UX (6.50 mg/kg + Al (4.2 mg/kg)	+++	++++	++++

**Table 7:** Electron microscopic findings in cortex and hippocampus regions of rat brains after treatment with boron compounds and Al. (- nonsignificant; + significant at %0.01; ++ significant at %0.1; +++ significant at %1; ++++ significant at %5, +++++ significant at 10%).

	Vacuolar Degeneration	Nuclear Pyknosis	Mitochondrial Damage	Number of Secondary Lysosomes
Control	-	-	-	-
Al (4.2 mg/kg)	+++++	+++++	+++++	+++++
BA (3.25 mg/kg)	-	-	-	-
BA (6.50 mg/kg)	-	-	-	-
BX (3.25 mg/kg)	-	-	-	-
BX (6.50 mg/kg)	-	-	-	-
Col (3.25 mg/kg)	-	-	-	-
Col (6.50 mg/kg)	-	-	-	-
UX (3.25 mg/kg)	-	-	-	-
UX (6.50 mg/kg)	-	-	-	-
BA (3.25 mg/kg) + Al (4.2 mg/kg)	++	++	+++	+++
BA (6.50 mg/kg) + Al (4.2 mg/kg)	++++	++++	++++	++++
BX (3.25 mg/kg) + Al (4.2 mg/kg)	++	+	++	++
BX (6.50 mg/kg) + Al (4.2 mg/kg)	++++	++++	++++	++++
Col (3.25 mg/kg) + Al (4.2 mg/kg)	+++	+++	+++	+++
Col (6.50 mg/kg) + Al (4.2 mg/kg)	++++	++++	++++	++++
UX (3.25 mg/kg) + Al (4.2 mg/kg)	+++	+++	+++	+++
UX (6.50 mg/kg + Al (4.2 mg/kg)	++++	++++	++++	++++

## Data Availability

The data presented in this study are available on request from the corresponding author. The data are not publicly available due to privacy.

## Supplementary Materials

The following are available online at https://www.mdpi.com/article/10.3390/toxics10080428/s1, Figure S1: In vivo effects of treatments with boron compounds and Al on (a) SOD, (b) CAT and (c) GSH-Px enzyme activities, and (d) MDA, (e) TAC and (f) TOS levels in rat serum. Symbol (*) represents statically significant difference compared to the Al-treated group.
