# Supplementary material for: Boron Compounds Exhibit Protective Effects against Aluminum-Induced Neurotoxicity and Genotoxicity: In Vitro and In Vivo Study
Source: Toxics. 2022 Jul 28;10(8):428. doi: 10.3390/toxics10080428 (PMC9413983; doi:10.3390/toxics10080428)
Supplement: Supplementary file 1 [file toxics-10-00428-s001.zip › toxics-1730081-supplementary.pdf]

toxics

MDPI

Supplementary Materials: Boron Compounds Exhibit Protective Effects against Aluminum-Induced Neurotoxicity and Genotoxicity: In Vitro and In Vivo Study

Hasan Turkez, Serkan Yıldırım, Elvan Sahin, Mehmet Enes Arslan, Bugrahan Emsen, Ozlem Ozdemir Tozlu, Gonca Alak, Arzu Ucar, Abdulgani Tatar, Ahmet Hacimuftuoglu, Mevlut Sait Keles, Fatime Geyikoglu, Muhammed Atamanalp, Fatih Saruhan and Adil Mardinoglu

(a)

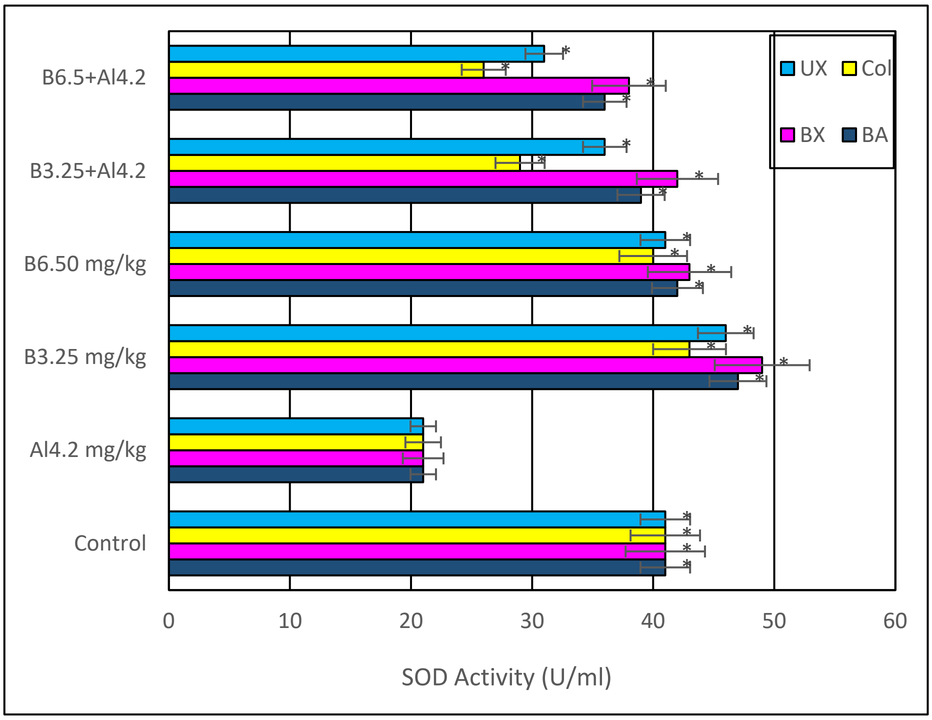

(b)

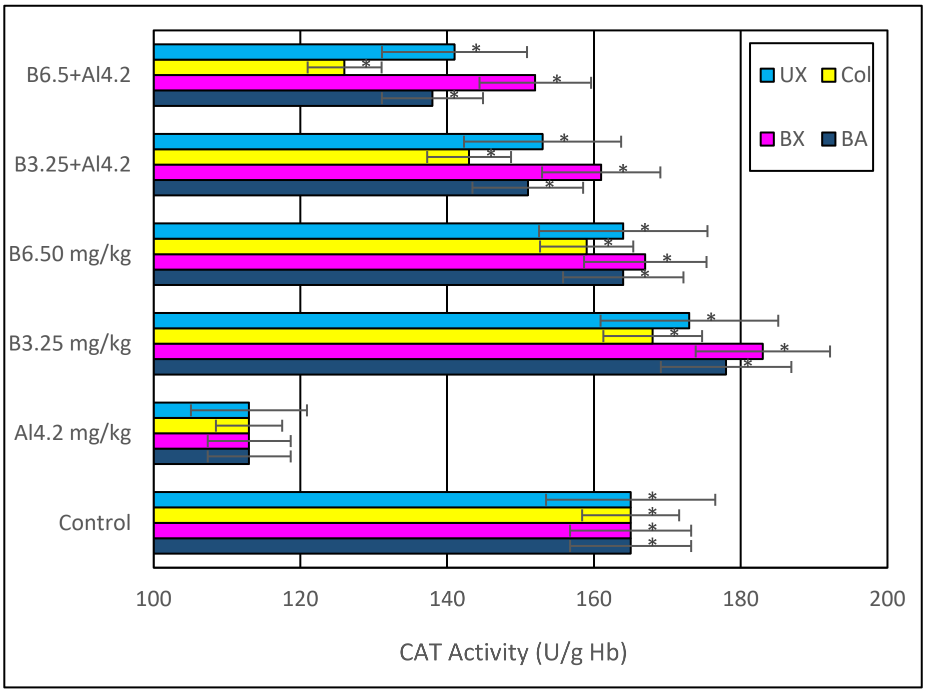
B6.5+Al4.2
B3.25+Al4.2
B6.50 mg/kg
B3.25 mg/kg
Al4.2 mg/kg
Control
UX
Col
BX
BA
100
120
140
160
180
200
CAT Activity (U/g Hb)

(c)

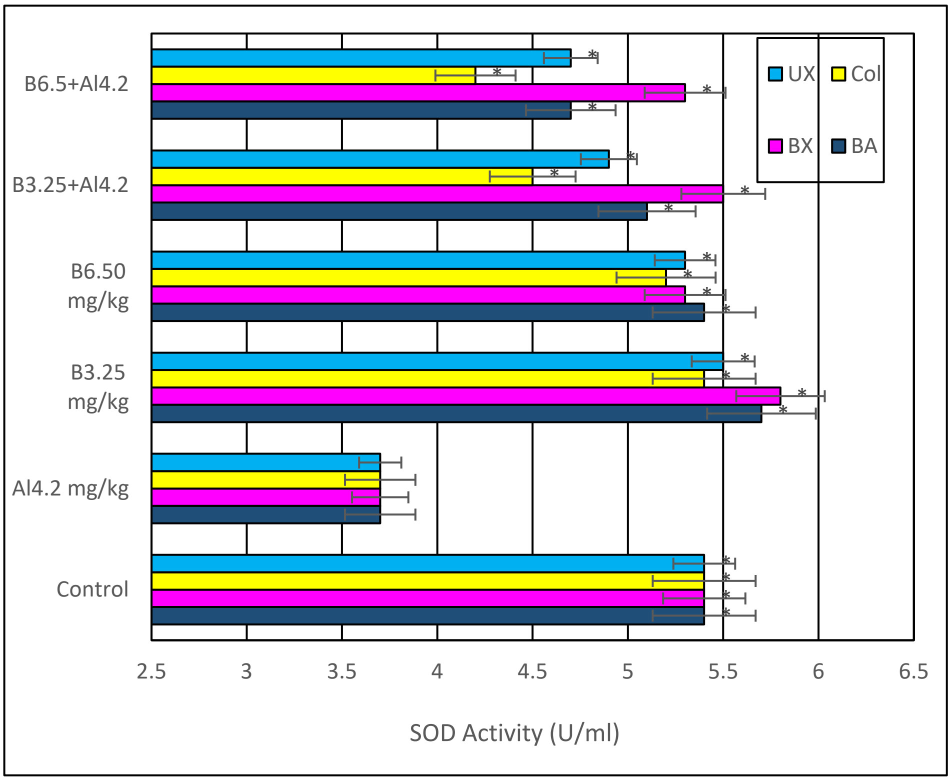
B6.5+Al4.2
B3.25+Al4.2
B6.50 mg/kg
B3.25 mg/kg
Al4.2 mg/kg
Control
UX
Col
BX
BA
2.5
3
3.5
4
4.5
5
5.5
6
6.5
SOD Activity (U/ml)

(d)

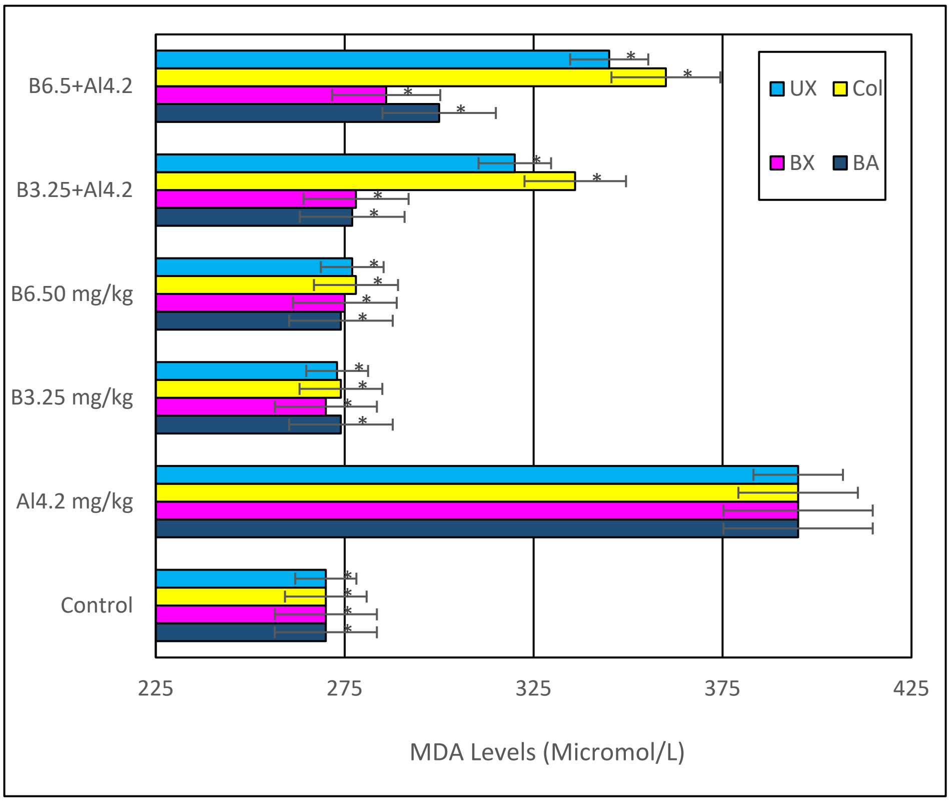
B6.5+Al4.2
B3.25+Al4.2
B6.50 mg/kg
B3.25 mg/kg
Al4.2 mg/kg
Control
UX
Col
BX
BA
225
275
325
375
425
MDA Levels (Micromol/L)

(e)

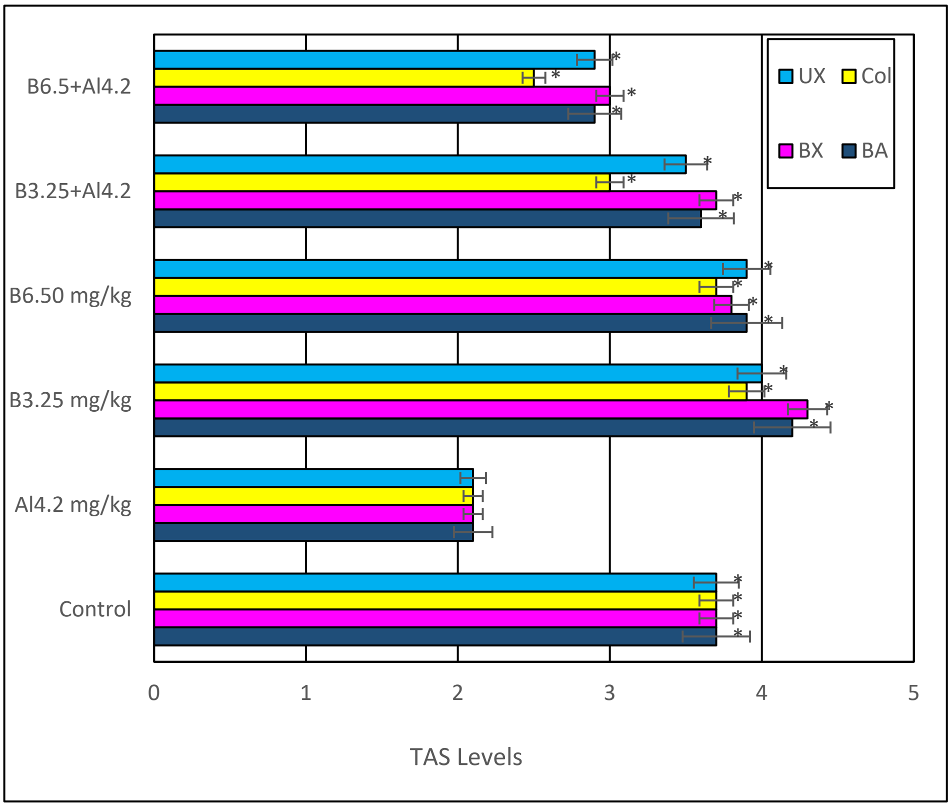
B6.5+Al4.2
B3.25+Al4.2
B6.50 mg/kg
B3.25 mg/kg
Al4.2 mg/kg
Control
UX
Col
BX
BA
0
1
2
3
4
5
TAS Levels

(f)

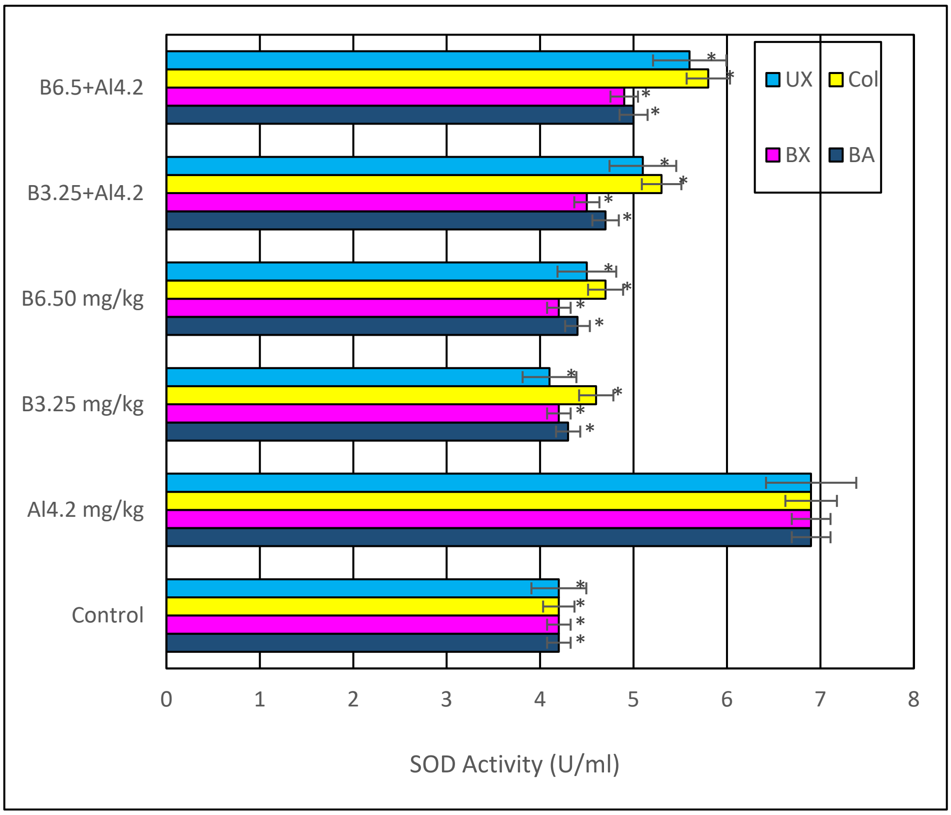

Figure S1. In vivo effects of treatments with boron compounds and Al on (**a**) SOD, (**b**) CAT and (**c**) GSH-Px enzyme activities, and (**d**) MDA, (**e**) TAC and (**f**) TOS levels in rat serum. Symbol (*) represents statically significant difference compared to the Al-treated group.
